# Integration of disease association and eQTL data using a Bayesian colocalisation approach highlights six candidate causal genes in immune-mediated diseases

**DOI:** 10.1093/hmg/ddv077

**Published:** 2015-03-05

**Authors:** Hui Guo, Mary D. Fortune, Oliver S. Burren, Ellen Schofield, John A. Todd, Chris Wallace

**Affiliations:** 1 JDRF/Wellcome Trust Diabetes and Inflammation Laboratory, Department of Medical Genetics, NIHR Cambridge Biomedical Research Centre, Cambridge Institute for Medical Research, University of Cambridge, Wellcome Trust/MRC Building, Cambridge Biomedical Campus, Cambridge CB2 0XY, UK,; 2 Centre for Biostatistics, Institute of Population Health, The University of Manchester, Jean McFarlane Building, Oxford Road, Manchester M13 9PL, UK and; 3 MRC Biostatistics Unit, Cambridge Institute of Public Health, Forvie Site, Robinson Way, Cambridge Biomedical Campus, Cambridge CB2 0SR, UK

## Abstract

The genes and cells that mediate genetic associations identified through genome-wide association studies (GWAS) are only partially understood. Several studies that have investigated the genetic regulation of gene expression have shown that disease-associated variants are over-represented amongst expression quantitative trait loci (eQTL) variants. Evidence for colocalisation of eQTL and disease causal variants can suggest causal genes and cells for these genetic associations. Here, we used colocalisation analysis to investigate whether 595 genetic associations to ten immune-mediated diseases are consistent with a causal variant that regulates, in *cis*, gene expression in resting B cells, and in resting and stimulated monocytes. Previously published candidate causal genes were over-represented amongst genes exhibiting colocalisation (odds ratio > 1.5), and we identified evidence for colocalisation (posterior odds > 5) between *cis* eQTLs in at least one cell type and at least one disease for six genes: *ADAM15*, *RGS1*, *CARD9*, *LTBR*, *CTSH* and *SYNGR1*. We identified cell-specific effects, such as for *CTSH*, the expression of which in monocytes, but not in B cells, may mediate type 1 diabetes and narcolepsy associations in the chromosome 15q25.1 region. Our results demonstrate the utility of integrating genetic studies of disease and gene expression for highlighting causal genes and cell types.

## Introduction

Genome-wide association studies (GWAS) have successfully identified many regions associated with a variety of diseases and other traits. However, the genes and cells that mediate these genetic associations remain only partially understood. Enrichment of disease-associated single-nucleotide polymorphisms (SNPs) amongst expression quantitative trait loci (eQTL) SNPs (1) indicates that a proportion of disease associations may be mediated through gene regulation. In the case where a disease association is mediated through its effect on gene expression, we would expect the eQTL and disease signal to occur at the same causal variant. However, as causal variants cannot be identified from genetic association data alone, showing that a single SNP is associated with both traits is not sufficient to confirm colocalisation, because the same situation could arise if two distinct causal variants were in linkage disequilibrium (LD) (2). We have previously used proportional colocalisation testing to show that *cis* eQTLs in resting monocytes are consistent with a shared causal variant for type 1 diabetes (T1D) in 21 genes across 14 T1D-associated regions (3). However, this analysis was limited by an inability to distinguish lack of statistical power from true colocalisation because the tested null hypothesis of colocalisation also corresponds to no association.

More recently, a Bayesian colocalisation method has been developed that assesses the support for multiple hypotheses and can distinguish lack of association from colocalisation or distinct associations (4). The Bayesian approach summarises evidence in a genetic region for five mutually exclusive hypotheses simultaneously:

H_0_: there exist no causal variants for either trait;
H_1_: there exists a causal variant for one trait only, disease;
H_2_: there exists a causal variant for one trait only, gene expression;
H_3_: there exist two distinct causal variants, one for each trait;
H_4_: there exists a single causal variant common to both traits.

Support for each is quantified in terms of posterior probabilities, denoted by PP_0_, PP_1_, PP_2_, PP_3_ or PP_4_, corresponding to the five hypotheses. In other words, posterior probabilities can be used to measure how likely each of the hypotheses are, given our prior beliefs of association and colocalisation, and the data on genetic association to disease and gene expression.

Here, we present the first application of Bayesian colocalisation analysis to integrate disease GWAS and eQTL data in order to highlight candidate causal genes and cells for ten immune-mediated diseases: autoimmune thyroid disease—ATD (5), celiac disease—CEL (6), Crohn's disease—CRO (7), multiple sclerosis—MS (8), narcolepsy—NAR (9), primary biliary cirrhosis—PBC (10), psoriasis—PSO (11), rheumatoid arthritis—RA (12), T1D (13) and ulcerative colitis—UC (7). Given the reported cell and state specificity of some eQTLs (14–16), it is important to consider eQTLs from primary immune cells, both resting and stimulated, and we used gene expression data from purified primary monocytes, both stimulated and resting (17,18), and resting B cells (19).

## Results

We considered a total of 595 published disease susceptibility regions for the ten immune-mediated diseases with summary association data available through http://www.immunobase.org (Table 1). Given the shared aetiology of many of these diseases, this corresponded to 154 non-overlapping regions. For each disease, dense genotype coverage is available through the ImmunoChip (20), a custom SNP microarray that provides common dense variant (SNP and small indels) coverage for regions associated with autoimmune and inflammatory diseases. We used gene expression from B cells (19), monocytes (17,18) and stimulated monocytes (interferon-γ after 24 h, IFN24; and lipopolysaccharide after 2 h, LPS2, and 24 h, LPS24) (17) to generate *cis* eQTL maps of 1414 unique genes within ±200 kb of these established disease susceptibility regions. For resting monocytes, we combined data from two studies in a fixed-effects meta-analysis. The samples in the gene expression studies had not been genotyped by the ImmunoChip, and so we imputed genotypes using 1000 Genomes Phase 1 haplotypes to provide a dense set of genotypes, as required by the colocalisation method. We used the Bayesian colocalisation analysis to examine evidence for colocalisation between disease and eQTL signals, for a total of 8369 pairwise comparisons.

**Table 1.** Number of case–control subjects and disease-associated regions for pairwise colocalisation investigation

Disease	Cases	Controls	Fairfax studies		Meta-analysis		Disease-eQTL.		
			Regions	Pairs	Regions	Pairs	Overlaps	Suggestive	Convincing
Autoimmune thyroid disease	2747	9364	9	125	8	65	6	2	0
Celiac disease	12 041	12 228	28	293	21	132	16	2	1
Crohn's disease	14 763	15 977	83	1110	77	619	61	5	2
Multiple sclerosis	9772	17 376	60	883	51	484	44	11	1
Narcolepsy	1886	10 421	2	19	2	6	1	1	1
Primary biliary cirrhosis	2861	8514	17	240	13	122	18	3	2
Psoriasis	10 588	22 806	24	423	22	253	15	1	0
Rheumatoid arthritis	13 838	33 742	60	905	58	536	11	0	0
Type 1 diabetes	6693	12 420	41	625	37	320	23	6	1
Ulcerative colitis	10 920	15 977	55	787	51	422	35	4	1
At least one disease							125	28	6
Total			379	5410	340	2959			

Probes within ±200 kb of the regions were included. ‘Regions’ gives the number of regions considered for each disease and ‘Pairs’ the number of probe/disease pairwise analyses performed. Given the different chips used for expression analyses, the number of regions differed between the Fairfax (17,19) and Cardiogenics (18) studies. The Disease-eQTL columns summarize the colocalisation analyses, giving the number of genes with a convincing eQTL overlapping a disease region (‘Overlaps’, PP_3_ + PP_4_ > 0.99), the number of those with suggestive evidence of colocalisation with the disease signal (‘Suggestive’; PP_4_/PP_3_ > 1) and the number of those with convincing evidence for colocalisation (‘Convincing’; PP_4_/PP_3_ > 5). The ‘Total’ row gives the total number of analyses performed (a simple sum of the numbers above). The ‘At least one disease’ row gives the number of genes showing eQTLs that overlap with or show suggestive or convincing colocalisation with at least one disease.

**Figure 1.**
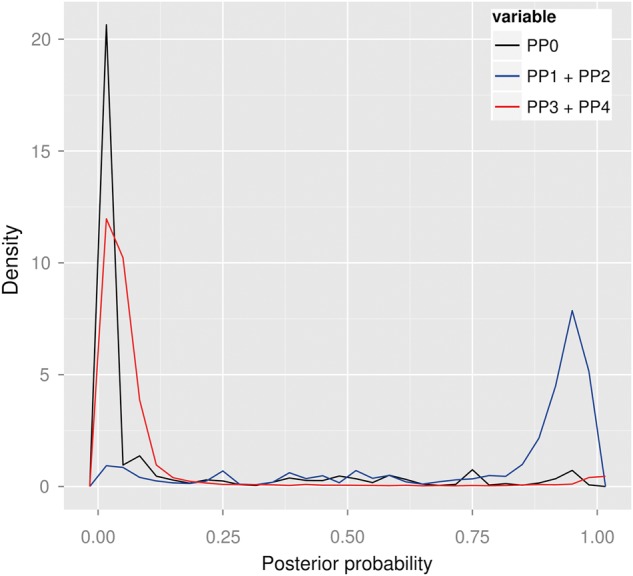
Overall results of colocalisation analyses. No evidence for association with either trait is found in most cases. Where association is observed, it is mostly with a single trait. Convincing evidence for colocalisation exists in a small proportion of genes. Shown are three posterior densities (PP_0_: causal variant for neither trait—black; PP_1_ + PP_2_: causal variant for one trait—blue; PP_3_ + PP_4_: causal variant(s) for both traits—red) of all pairwise comparisons performed between the diseases and gene expression (unstimulated monocytes and B cells, stimulated monocytes after 24-h IFN, 2-h LPS and 24-h LPS).

Figure 1 shows the densities of the posterior probabilities of no association for either trait (PP_0_), association with exactly one trait (PP_1_ + PP_2_) and association with both traits (PP_3_ + PP_4_). In most cases, no evidence for association is found with either trait and, where association is observed, it is mostly with a single trait. Restricting attention to 1239 probe/disease pairs with PP_3_ + PP_4_ ≥ 0.8 (suggestive of association with both traits), published candidate causal genes are over-represented amongst pairs exhibiting colocalisation (odds ratio > 1, Table 2), suggesting that colocalising signals identify disease-relevant genes in all cells and conditions analysed. A total of 125 genes showed eQTL signals that overlapped with at least one disease (PP_3_ + PP_4_ ≥ 0.99, Table 1). Of these, 28 also showed some support for colocalising signals (PP_4_/PP_3_ > 1) but only six showed sufficient support for us to consider them ‘convincing’ (PP_4_/PP_3_ > 5, Table 3). The number of colocalising signals found reflects the sample numbers available in the eQTL studies, with more pairwise colocalising signals found in resting monocytes (*n* = 9) than in stimulated monocytes (*n* = 6) or B cells (*n* = 4). A further three genes showed weaker, but still supportive evidence (PP_4_/PP_3_ > 0.9 and PP_4_/PP_3_ > 3, Table 3). All of these have previously been nominated as candidate causal genes through association of expression of disease-associated SNPs: *BLK* in RA through expression in B cells (21), as here, *FAM119B* as a candidate causal gene in MS through expression in whole blood (22), matching the colocalisation we observe across B cells and monocytes, and *FAM164A* through expression in lymphoblastoid cell lines (23). Supplementary Material, Figures S1–S11 show the zoomed Manhattan plots for implicated genes and diseases in Table 3.

**Table 2.** Colocalisation is over-represented amongst probe/disease pairs in published candidate causal genes across all five cell types (odds ratio > 1)

Cell type	In candidate genes		Not in candidate genes		Odds ratio	*P*
	Pairs	Odds	Pairs	Odds		
Resting cells						
B cells	63	0.167	94	0.106	1.575	0.514
Monocytes	92	0.195	267	0.077	2.532	0.017
Stimulated monocytes						
IFN, 24 h	99	0.165	146	0.074	2.230	0.096
LPS, 2 h	46	0.179	127	0.085	2.106	0.252
LPS, 24 h	50	0.163	133	0.099	1.646	0.477

‘Pairs’ gives the number of probe/disease pairs with evidence of association with both traits (PP_3_ + PP_4_ > 0.8) and ‘Odds’ the odds that a pairwise comparison gives greater posterior support to a shared causal variant (PP_4_ > PP_3_). Published candidate causal genes are as curated in ImmunoBase.

**Table 3.** Six genes showing evidence for colocalisation between gene expression and disease with PP_3_ + PP_4_ ≥ 0.99 and PP_4_/PP_3_ ≥ 5 and three genes with weaker evidence (PP_3_ + PP_4_ ≥ 0.90 and PP_4_/PP_3_ ≥ 3)

Region	Disease	Gene	Probe	B cells		Monocytes		Mono + 24-h IFN		Mono + 2-h LPS		Mono + 24-h LPS		Dir.
				Assoc	Coloc	Assoc	Coloc	Assoc	Coloc	Assoc	Coloc	Assoc	Coloc	
**PP_3_ + PP_4_ ≥ 0.99 and PP_4_/PP_3_ ≥ 5**														
1q22	CRO	*ADAM15*	ILMN_1751500	0.054	0.218	**0.998**	**22.158**	**0.998**	**22.055**	**0.998**	**22.158**	**0.998**	**22.158**	†
* 1q31.2	CEL	*RGS1*	ILMN_1656011	**1.000**	**6.407**	**1.000**	**3.739**	0.312	3.168	0.013	0.839	0.476	4.672	−
* 1q31.2	MS	*RGS1*	LMN_1656011	**1.000**	**5.369**	**1.000**	**3.167**	0.330	2.806	0.015	0.693	0.482	4.021	−
9q34.3	CRO	*CARD9*	ILMN_1712532	0.062	0.125	**1.000**	**1.890**	**1.000**	**1.370**	**1.000**	**6.246**	**1.000**	**3.831**	+
9q34.3	UC	*CARD9*	ILMN_1712532	0.067	0.128	**1.000**	**1.976**	**1.000**	**1.597**	**1.000**	**11.956**	**1.000**	**6.519**	+
12p13.31	PBC	*LTBR*	ILMN_1667476	0.010	1.426	**1.000**	**8.524**	**0.999**	**3.647**	**1.000**	**4.435**	**1.000**	**4.650**	+
15q25.1	NAR	*CTSH*	ILMN_2390853	*0.999*	*0.001*	**1.000**	**99.000**	**1.000**	**96.117**	**1.000**	**95.192**	**1.000**	**99.000**	−
15q25.1	T1D	*CTSH*	ILMN_2390853	*1.000*	*0.001*	**1.000**	**32.013**	**1.000**	**141.453**	**1.000**	**303.040**	**1.000**	**32.333**	−
22q13.1	PBC	*SYNGR1*	ILMN_1727805	**1.000**	**13.925**	**1.000**	**11.956**	0.020	0.350	0.024	0.606	0.060	0.392	±^$^
22q13.1	PBC	*SYNGR1*	ILMN_1810875	**1.000**	**16.602**	*1.000*	*0.000*	0.681	0.007	0.193	0.095	0.831	0.002	+
**PP_3_ + PP_4_ ≥ 0.90 and PP_4_/PP_3_ ≥ 3**														
8p23.1	RA	*BLK*	ILMN_1668277	**0.909**	**5.184**	*0.022*	*0.355*	0.030	0.212	0.100	2.752	0.032	0.633	−
8q21.12	MS	*FAM164A*	ILMN_1789558	**1.000**	**3.525**	**1.000**	**3.115**	**1.000**	**3.608**	**1.000**	**2.891**	**1.000**	**3.167**	−
8q21.12	MS	*FAM164A*	ILMN_2057981	**1.000**	**3.348**	**1.000**	**3.310**	**1.000**	**3.202**	0.896	2.343	**1.000**	**2.984**	−
12q14.1	MS	*FAM119B*	ILMN_1723846	**1.000**	**3.566**	**1.000**	**4.051**	**1.000**	**3.717**	**1.000**	**3.329**	**1.000**	**2.311**	−

Assoc is the posterior probability that both the disease is associated with the region and that an eQTL exists in the indicated cell type. Coloc is the posterior odds of colocalisation, given that both traits are associated. When data for a given probe were available from both studies of monocyte expression, we present the results using a meta-analysis of the two. When data were only available from one study (17), we used just that study, indicated by ‘*’. Evidence of colocalising effects is indicated by bold font, evidence of distinct effects by italic font. Allele includes risk (left) and protective alleles (right). Dir. shows whether disease risk across the associated SNPs in a region correlates with increased (+) or decreased (−) mRNA expression of the gene. † is a secondary disease signal, and direction of effect was not published for Crohn's disease. ^$^For 22q13.1/*SYNGR1*, the PBC risk allele correlated with increase expression in B cells (B) but decreased expression in monocytes (M).

For each region in which at least one colocalising signal was observed, we examined the evidence for other probes, genes and diseases in that region (Supplementary Material, Figs S12–S20). No colocalised signals were observed convincingly across all cell types and conditions, emphasizing the cell- and condition-specific effects of disease causal variants. For example, colocalisation between reduced *CTSH* expression and T1D and NAR susceptibility on chromosome 15q25.1 was seen in both resting and stimulated monocytes but not in B cells. The monocyte eQTL (peak SNP, rs34843303) appears to also affect expression in B cells, but there is a stronger eQTL signal unique to B cells at SNP rs11855406 (Supplementary Material, Fig. S5). This suggests that disease susceptibility may be mediated through monocytes or through another cell type with an equivalent eQTL pattern, but not through B cells, at least under this condition.

**Figure 2.**
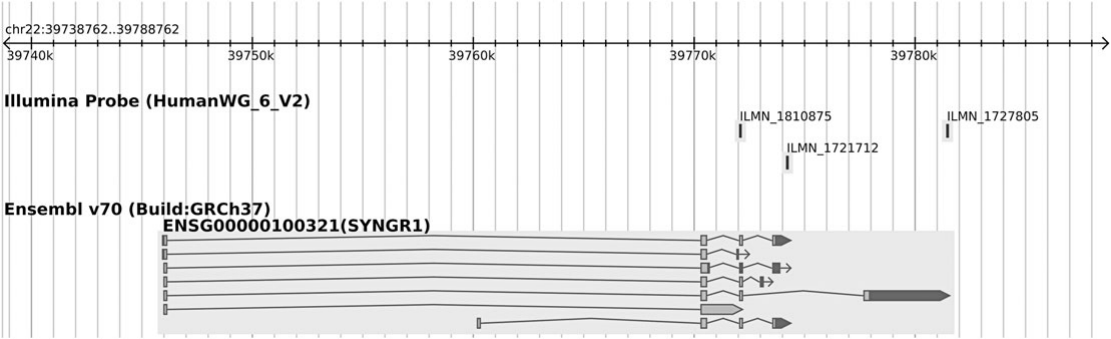
In the gene *SYNGR1*, expression of ILMN_1810875 is captured by almost all isoforms but that of ILMN_1727805 is captured by only one isoform. Shown is gene context plot of *SYNGR1* depicting the positions of three probes.

The design of the expression microarray occasionally includes multiple probes for a single gene in an attempt to capture isoform-specific expression. We observed colocalisation between PBC association marked by rs2267407 and eQTLs for *SYNGR1* in resting monocytes and B cells using the probe ILMN_1727805, but colocalisation with only the B cell eQTL using the probe ILMN_1810875. No convincing eQTLs were seen in stimulated cells. ILMN_1727805 captures a single isoform whereas ILMN_1810875 captures almost all isoforms (Fig. 2), suggesting that there may be a monocyte-specific eQTL that does not affect expression of the long isoform captured by ILMN_1727805, and that, therefore, perhaps only the long isoform of *SYNGR1* is involved in the aetiology of PBC.

## Discussion

**Figure 3.**
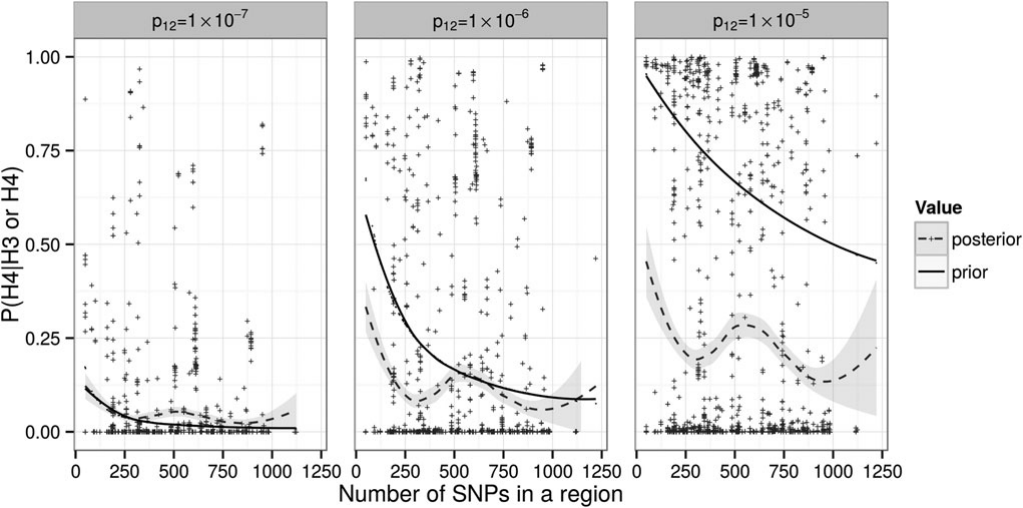
Effect of prior probability *p*_12_ on the prior and posterior support for colocalisation in regions where both disease and eQTL show evidence for association (PP_3_ + PP_4_ > 0.8). We repeated the colocalisation analysis with all prior probabilities fixed as specified in Materials and Methods, except *p*_12_ which varied between 10^−7^ and 10^−5^. For each pairwise analysis in which PP_3_ + PP_4_ > 0.8, we plotted the number of SNPs in that region against the relative posterior support for H_4_, defined by PP_4_/(PP_3_ + PP_4_) (green), and similarly the relative prior support (orange).

In the Bayesian colocalisation approach adopted for our analyses, selection of prior beliefs for each hypothesis plays an important role. Giambartolomei *et al*. (4) assumed a random SNP might be causal for either trait individually with prior probability 1 × 10^−4^. We adopted the same value here as it equates to a conservative expectation of ∼20 causal SNPs on the ImmunoChip array for any disease and ∼1 in 20 genes having a *cis* eQTL in a given cell type. Given that we are focusing on regions already known to show genome-wide association to disease, this prior for disease association may appear low: within any region we consider, we do expect there to exist a causal variant. However, the samples used to declare genome-wide association in each region are generally the same as, or a subset of, those used to generate the summary statistics used in our analysis, and we cannot alter a prior belief on the basis of the same data we are about to analyse. Giambartolomei *et al*. then considered prior probabilities for an SNP being causal for both traits (*p*_12_) ranging from 1 × 10^−5^ to 1 × 10^−6^ and stated that consistent results were shown given those different priors. However, for our dataset, we found results depended quite substantially on the prior (Fig. 3). Whilst the interpretation of the priors for association of each individual trait is clear and we have considerable data amassed through GWAS and eQTL studies with which to support our choice, there is substantially less data quantifying the role of eQTLs in mediating GWAS hits to guide our choice of *p*_12_. We used a form of internal empirical calibration (see Materials and Methods), considering a range of values for *p*_12_ and choosing that for which the posterior expectation of colocalisation, averaged over all regions considered, most closely resembled the prior expectation of colocalisation. We encourage other researchers to consider carefully the parameters used to define the prior probabilities as they may vary according to the cell type or diseases under study.

Figure 3 also reflects the dependence of the prior probability of colocalisation on the number of SNPs within a region supposed to harbour causal variants for disease and gene expression. This is perhaps not entirely intuitive for researchers used to thinking in terms of single SNPs rather than regions. If a region contains *Q* SNPs, then any one of them may be causal for each trait, and, given our assumption that one SNP is causal for each trait, the chance of the same SNP being causal for both decreases as *Q* increases. Thus, our prior probability of colocalisation is, appropriately, lower in regions which contain a greater number of variants.

Our results do not capture all the previous candidate causal genes that have been suggested in previous analysis of these eQTL datasets. There are a number of reasons for this. First, because the colocalisation method requires dense genotyping, we restricted attention to the densely genotyped target regions on ImmunoChip, which cover ∼1% of the genome. Although enriched, by design, for regions harbouring autoimmune disease variants, they do not contain associations that were published after the design of the ImmunoChip in 2009/2010. Second, we have been very stringent in declaring a colocalised signal. We required genome-wide significance in the disease data, *P* < 10^−10^ in the eQTL data, and strong evidence for colocalisation (PP_4_*/*PP_3_ > 5). Whilst we provide complete results in the supplementary material that will be useful for researchers wishing to perform further confirmatory analysis, we decided to focus in the main text only on those most convincing results. Third, the colocalisation method requires that the strongest signals in a region colocalise. Where there are primary and secondary eQTL signals, we do not declare colocalisation if the disease signal matches the secondary signal alone. For example, a secondary eQTL with *IL18R1* in monocytes after 2 h of LPS stimulation has been linked with the celiac disease signal on chromosome 2q12.1 (17). Supplementary Material, Figure S21 highlights the primary and secondary eQTL signals (tagged by rs4851572 and rs990171, respectively, with the two SNPs in weak LD: *r*^2^ = 0.3) and shows that only the secondary signal can be seen for celiac disease. How such colocalising signals should be interpreted is not clear. The secondary signal is likely to be relevant for both celiac disease, and the expression of *IL18R1* in LPS-stimulated monocytes, but *IL18R1* expression in LPS-stimulated monocytes cannot itself mediate the celiac disease association in the region, otherwise any SNP that controlled expression of *IL18R1* would also associate with celiac disease. Such results therefore, in our opinion, highlight a gene but not a gene and its disease-relevant cell and state, and it is probable that a more specific stimulation condition might reveal a completely colocalised signal between *IL18R1* expression and celiac disease. A similar situation is seen for *CTSH* expression in B cells and NAR and T1D susceptibility on chromosome 15q25.1, whereas colocalisation with *CTSH* expression in monocytes is clear, with both signals tagged by SNP rs34843303 (Supplementary Material, Fig. S5).

Whilst this third reason is essentially a difference of interpretation, the fourth reason that our results can differ from previous reports is that formal colocalisation uses more information and can therefore provide a more complete picture, than simply looking for overlaps between GWAS and eQTL results. This emphasises the need to apply a formal colocalisation analysis. For example, an eQTL with *PDGFB* in monocytes after 2 h of LPS stimulation has been linked with PBC susceptibility on chromosome 22q13.1 (17). We found no evidence for colocalisation in LPS-stimulated monocytes (PP_4_/PP_3_ = 0.1) but instead obtained evidence for colocalisation with expression of *SYNGR1* in B cells (PP_4_/PP_3_ = 16.6). Examining the Manhattan plots (Supplementary Material, Fig. S22) shows both the PBC signal and the *SYNGR1* eQTL are tagged by rs2267407 and the *PDGFB* eQTL tagged by rs968451. The two SNPs are in LD (*r*^2^ = 0.71), but the patterns of association across the region clearly support *SYNGR1* expression in B cells rather than *PDGFB* expression in LPS-stimulated monocytes as a disease colocalising signal. However, neither gene has obvious functional candidacy, and the original GWAS report highlighted *MAP3K7IP1* as a candidate gene (24).

Of particular note, we did not detect strong evidence for any colocalising signals across all studied cell types and states. For example, we identified a disease colocalising eQTL for *CARD9* and the diseases CRO and UC only in monocytes after LPS stimulation, as previously reported (17). Whilst there is evidence for an eQTL in all monocyte states, and the odds favour colocalisation in each, the evidence for colocalisation is strong only after LPS stimulation. This emphasizes that disease causal variants are likely to act in cell and/or state specific mechanisms and motives the collection of large, publicly available eQTL datasets for a wider range of purified cell types and activation states that might be more relevant to disease at the site of pathology, to enable researchers to more completely identify the mechanisms underlying genetic susceptibility to disease. Despite this, when we looked for response eQTLs (those that determine the difference between baseline and stimulated expression, rather than those that determine expression either at baseline or stimulation), we did not find any colocalising signals. This is likely to relate to issues of correctly normalising expression measurements between two samples (stimulated and unstimulated). Other colocalising signals were specific to individual probes, and therefore, could indicate isoform-specific effects. This also indicates a need to better measure expression at the isoform rather than gene level, which is becoming possible using RNA sequencing (25).

One of the strongest colocalising signals we observe is that of monocyte *CTSH* expression and T1D and NAR risk in the 15q25.1 region. *CTSH* is a member of the family of cathepsins and is involved in apoptosis and antigen presentation, amongst other roles. The peak *CTSH* eQTL SNP we find in B cells is rs11855406, which is in LD (*r*^2^ = 0.96) with a previously reported T1D-associated SNP in the region, rs3825932:T>C (26). The major, T1D predisposing, allele of rs3825932, T, has been shown to correlate with decreased *CTSH* mRNA and protein expression in human B-lymphoblastoid cell lines (27), and pro-inflammatory cytokines have been shown to reduce expression of *CTSH* in human pancreatic islets where these cytokines cause stress and increased apoptosis of islet beta cells (27).

Using the denser SNP map provided by ImmunoChip, the peak of T1D association in this region now resides at rs34843303 and aligns with that for NAR. We have shown here that the eQTL signal in B cells is not compatible with T1D and NAR risk being mediated by *CTSH* expression in B cells but that in both resting and stimulated monocytes is (Table 3, Supplementary Material, Fig. S5). Autoimmune diseases are due to the inappropriate targeting by the immune system of host tissue, and there has been debate, particularly with regard to T1D, as to the relative contribution of each in the aetiology of disease. NAR is not known to involve pancreatic islets, and the joint colocalisation of T1D and NAR association signals with *CTSH* expression by monocytes suggests that the effects of *CTSH* reported by Fløyel *et al*. (27) in islets imply that this gene might exert its effect on T1D by variable expression in multiple cell and tissue types.

The other strongest signal we observe links monocyte *ADAM15* expression and Crohn's disease risk in the 1q22 region. *ADAM15* is a metalloprotease, a member of the ADAM (a disintegrin and metalloproteinase) protein family that includes *ADAM30*, an existing candidate gene for Crohn's disease (28). *ADAM15* has been shown to be upregulated in colon tissue from inflammatory bowel disease patients compared with healthy controls (29). Similarly, in our data, the minor allele of rs11589479 is associated with increased disease risk and increased expression of *ADAM15*. However, without knowing the allele-specific expression of *ADAM15* in colon tissue, we cannot determine whether our colocalisation results reflect a similar eQTL profile in monocytes and colon, or whether expression of *ADAM15* in tissue resident monocytes may itself increase inflammation at the site of disease in patients with Crohn's disease.

The relatively low rate of convincing colocalisation in our results does not indicate, in our opinion, that variation in gene expression is not a substantial mediator of genetic effects on disease risk. Rather, it is likely to reflect the sample size limitations of existing available eQTL studies that are focused on a subset of accessible cells, mostly from peripheral blood, and which are still only of moderate sample sizes. Larger sample sizes and a wider variety of purified cells in resting and activated states are needed to more completely assess the role that variation in gene expression plays in mediating genetic risk of disease. Ultimately, specific functional studies will be required to confirm or refute the candidacy of the genes we highlight here. Nevertheless, whilst an estimated 50% of GWAS hits lie distal to genes (30), eQTL-disease colocalisation analyses provide a means to integrate genetic and gene expression data to highlight directions for further study.

## Materials and Methods

### Case–control data

All the case–control subjects were genotyped using the ImmunoChip, an Illumina 200K Infinium high-density array. Summary statistics, including *P*-values and MAF, were downloaded from ImmunoBase (http://www.immunobase.org, accessed 04 May 2014). The number of participants for each disease is shown in Table 1. Samples were of European ancestry and are described in detail in the original papers (5–13).

### eQTL data

The eQTL study of monocytes (17) comprises 414 (unstimulated), 367 (IFN24), 261 (LPS2) and 322 (LPS24) volunteers of European ancestry recruited in the Oxfordshire area (gene expression data: http://www.ebi.ac.uk/arrayexpress/experiments/E-MTAB-2232; genotyping data: http://www.ebi.ac.uk/ega/EGAD00010000144, http://www.ebi.ac.uk/ega/EGAD00010000520). The eQTL study of B cells (19) consists of 288 volunteers, of whom approximately two-thirds overlap with the subjects in the monocytes study, from the same area (gene expression data: http://www.ebi.ac.uk/arrayexpress/experiments/E-MTAB-945; genotyping data: http://www.ebi.ac.uk/ega/EGAD00010000144). We included 413 (unstimulated), 366 (IFN24), 260 (LPS2) and 321 (LPS24) subjects that have per-sample call rate of >0.92 and autosomal heterozygosity of >0.3225.

Samples of the Cardiogenics monocytes eQTL study (https://www.ebi.ac.uk/ega/studies/EGAS00001000411) were European descent and were recruited in five centres. We restricted analysis to 396 non-diabetic subjects who had genetic and expression data of monocytes available in the study. After per-sample call rate (=1) and autosomal heterozygosity (≥0.335 and ≤0.346) filtering, 391 remained for further analyses.

Samples from the Fairfax studies were genotyped using the Illumina HumanOmniExpress-12v1.0 BeadChip. Samples from the Cardiogenics study were genotyped using Human 610 Quad Custom arrays. To provide matching dense genotypes in eQTL samples, we imputed genotypes into 1000 Genomes Phase 1 haplotypes using IMPUTE2 (31) (http://mathgen.stats.ox.ac.uk/impute/impute_v2.html), for all the disease-associated regions.

### Disease-associated regions

Amongst the up-to-date disease-associated regions (http://www.immunobase.org/downloads/regions-files-archives/2014-05-04, archived on 04 May 2014), 545 unique non-MHC autosomal regions were initially included and 341 regions were used for colocalisation analysis after multi-stage quality control.

### eQTL probeset, SNP and region quality control

For probes including multiple position windows, we used minimum and maximum values of the positions to define their coordinates. As causal variation may well affect regulatory regions, we tested for colocalisation for probes in neighbouring genes within ±200 kb of the disease-associated regions. Although regulatory regions may lie at a considerable distance from their target genes, most *cis* eQTLs are thought to lie within 100 kb of their target gene (32). We took a pragmatic decision to use a 200-kb window to balance our desire to capture likely *cis*-mediating disease genes and the need to limit any multiple testing.

Genotyped SNPs were filtered based on minor allele frequencies MAF (≥0.005), per-SNP call rate (≥0.99) and *z*-score of Hardy–Weinberg equilibrium (|*z*| ≤ 4). The imputed SNPs with poor quality [MAF < 0.005, |*z*| > 4 and the imputation output ‘info’ (similar to *R*^2^ metrics) <0.3] were also removed. Based on the table (Annotation Illumina Human-WG-V3-controls_hg18_V1.0.0_Aug09.txt) provided in ReMOAT (33) (http://remote.sysbiol.cam.ac.uk/sequence.php), probes with poor quality scores were excluded. After imputation, we excluded the autosomal regions that had no probes within ±200 kb, or <20 common SNPs with the diseases.

Because the genome assembly of the eQTL data was NCBI36, whilst that of the Immunochip data as curated in ImmunoBase and the 1000 Genomes data were GRCh37, we converted the assembly of the SNPs and coordinates of the probes of the eQTL study to GRCh37 (http://genome.ucsc.edu/cgi-bin/hgLiftOver), before identifying valid probes and common SNPs with the GWAS.

Probes which indicated colocalising results were checked for the presence of overlapping SNPs using ReMOAT (http://remoat.sysbiol.cam.ac.uk/sequence.php). Signals where our peak SNP was in LD (*r*^2^ > 0.6) with any overlap SNP were excluded, as these may relate to the effect of the SNP on probe binding, rather than be a true reflection of gene expression.

### Colocalisation analyses

Colocalisation analyses were conducted using the R package coloc, http://cran.r-project.org/web/packages/coloc (4). The method requires summary statistics for each SNP: either *P*-values and MAFs, or estimated genotype effects and their standard errors. As genotypes were available for the eQTL data, we were able to compute the estimated effects and standard errors (snp.rhs.estimates, package snpStats, http://www.bioconductor.org). For the ten studied diseases, their *P*-values and MAFs from 1000 Genomes were used as input. The assumptions of the colocalisation analyses are as follows:

1. The pair of traits for colocalisation are from independent studies. Our tests for colocalisation between the expression of eQTL and the autoimmune diseases satisfy this assumption.
2. At most one SNP is causal for either of the traits in each test region. Under this assumption, we have five hypotheses: association with neither H_0_; with either (one of the ten diseases H_1_ or monocytes/B cells H_2_) and with both (at two independent SNPs H_3_, at one shared SNP H_4_). It is possible that more than one SNP is causal for a trait in a test region. However, in the probable case that one effect is stronger than another, the method effectively tests colocalisation of the strongest effect(s) (4).
3. The probability of that a given SNP is causal for a trait is independent of the probability that any other given SNP in the region is causal. This is perhaps counter-intuitive: we might expect the probability of a given SNP is causal is increased when another SNP is known to be causal. However, because we condition on exactly one or zero causal variants, these higher order probabilities need never be used. This allows the probability of each hypothesis to be expressed as shown in Equations (1)–(5) below. Note that this may not apply to association relationships, as associations of two or more SNPs with the trait will not be independent of each other if the SNPs are in high LD. However, it is plausible for causality.
4. Causal SNPs are either directly genotyped, very well tagged or well imputed. In the high-density regions of the ImmunoChip, imputation of common variants has >95% efficiency (34), justifying this assumption.

Under the above-mentioned assumptions, we set our models as follows. Suppose we have *Q* SNPs in a test region. Let *p*_0_, *p*_1_, *p*_2_, *p*_12_ denote the prior probabilities of an SNP being causal for neither trait, disease only, gene expression of the monocytes/B cells only and both, respectively. These four prior probabilities must sum up to 1 because they comprise all possible outcomes.

### Selection of prior

The prior probabilities corresponding to the five hypotheses are as follows:

(1) $$P_{0}=P(H_{0})=p_{0}^{Q};$$

(2) $$P_{1}=P(H_{1})=Q\times p_{0}^{Q-1}\;p_{1};$$

(3) $$P_{2}=P(H_{2})=Q\times p_{0}^{Q-1}\;p_{2};$$

(4) $$P_{3}=P(H_{3})=Q(Q-1)\times p_{0}^{Q-2}\;p_{1}\;p_{2};$$

and

(5) $$P_{4}=P(H_{4})=Q\times p_{0}^{Q-1}\;p_{12}.$$

Then, we have that, from Equations (4) and (5), the ratio of *P*_4_ (prior probability of shared causal variant) and *P*_3_ (prior probability of distinct causal variants):

(6) $$P_{4}/P_{3}=P(H_{4})/P(H_{3})=\frac{\;p_{0}\;p_{12}}{(Q-1)\;p_{1}\;p_{2}}.$$

Both *p*_1_ and *p*_2_ are set to 1 × 10^−4^. In other words, our belief from previous experience is that 1 in 10 000 SNPs is causal to either trait. Obviously, *p*_12_ should be smaller than *p*_1_ or *p*_2_ because it is less likely for an SNP to be causal for both traits. Consequently, *p*_0_ is approximately equal to 1, and therefore,

(7) $$P_{4}/P_{3}\approx \frac{10^{8}\;p_{12}}{\left(Q-1\right)}.$$

Hence, for a given region where the number of SNPs *Q* is fixed, *P*_4_/*P*_3_ is proportional to *p*_12_ and decreases as the number of SNPs in a region increases. Likewise, it can be shown that the ratio of posterior probabilities PP_4_/PP_3_ is proportional to *p*_12_. Moreover, both the sums *P*_4_ + *P*_3_ and PP_4_ + PP_3_ increase as *p*_12_ increases. To determine a sensible value for *p*_12_, we compared the prior and posterior relative probabilities for H_4_|H_3_ or H_4_ for three choices of *p*_12_: 10^−7^, 10^−6^ and 10^−5^ (Fig. 3). Empirically, the optimal choice of *p*_12_ should produce a similar expectation of H_4_/H_3_ or H_4_ under prior or posterior when the expectation is taken across all pairwise analyses. From graphical examination of these results, we chose *p*_12_ = 10^−6^ as the most appropriate value, i.e. we expect that one in 101 SNPs causal for at least one trait is causal for both traits.

## Supplementary Material

Supplementary Material is available at *HMG* online.

## Funding

This work was supported by the JDRF (9-2011-253), the Wellcome Trust (091157) and the National Institute for Health Research (NIHR) Cambridge Biomedical Research Centre. The research leading to these results has received funding from the European Union′s 7th Framework Programme (FP7/2007-2013) under grant agreement no.241447 (NAIMIT). The Cambridge Institute for Medical Research (CIMR) is in receipt of a Wellcome Trust Strategic Award (100140). The Wellcome Trust funded C.W. and H.G. (089989) and M.D.F. (099772). ImmunoBase.org is supported by Eli Lilly and Company. Funding to pay the Open Access publication charges for this article was provided by the Wellcome Trust.

## Supplementary Material

Supplementary Data
