# Supplementary material for: Integration of disease association and eQTL data using a Bayesian colocalisation approach highlights six candidate causal genes in immune-mediated diseases
Source: Hum Mol Genet. 2015 Mar 5;24(12):3305–13. doi: 10.1093/hmg/ddv077 (PMC4498151; doi:10.1093/hmg/ddv077)
Supplement: Supplementary Data [file supp_24_12_3305__index.html]

Integration of disease association and eQTL data using a Bayesian colocalisation approach highlights six candidate causal genes in immune-mediated diseases — Integration of disease association and eQTL data using a Bayesian colocalisation approach highlights six candidate causal genes in immune-mediated diseases — Integration of disease association and eQTL data using a Bayesian colocalisation approach highlights six candidate causal genes in immune-mediated diseases — Supplementary Data 

# Integration of disease association and eQTL data using a Bayesian colocalisation approach highlights six candidate causal genes in immune-mediated diseases

## Supplementary Data

Supplementary Data

**Files in this Data Supplement:**

- Supplementary Data - Pdf file
