# Supplementary material for: Integration of disease association and eQTL data using a Bayesian colocalisation approach highlights six candidate causal genes in immune-mediated diseases
Source: Hum Mol Genet. 2015 Mar 5;24(12):3305–13. doi: 10.1093/hmg/ddv077 (PMC4498151; doi:10.1093/hmg/ddv077)
Supplement: Supplementary Data [file supp_ddv077_ddv077supp.pdf]

Integration of disease association and eQTL data indicates seven candidate causal genes and highlights the role of monocytes in immune-mediated diseases
Supplementary Material

Hui Guo, Mary D Fortune, Oliver S Burren, Ellen Schofield, Neil M Walker, John A Todd, Chris Wallace

February 17, 2015

1 Manhattan plots

Each plot shows the $-\log_{10}(p)$ values for disease(s) and expression of gene(s) with evidence of association to each region. To allow visual resolution of distinct signals, points are coloured according to the LD (r^2) between the SNP they represent and the index SNP(s) shown in the key.

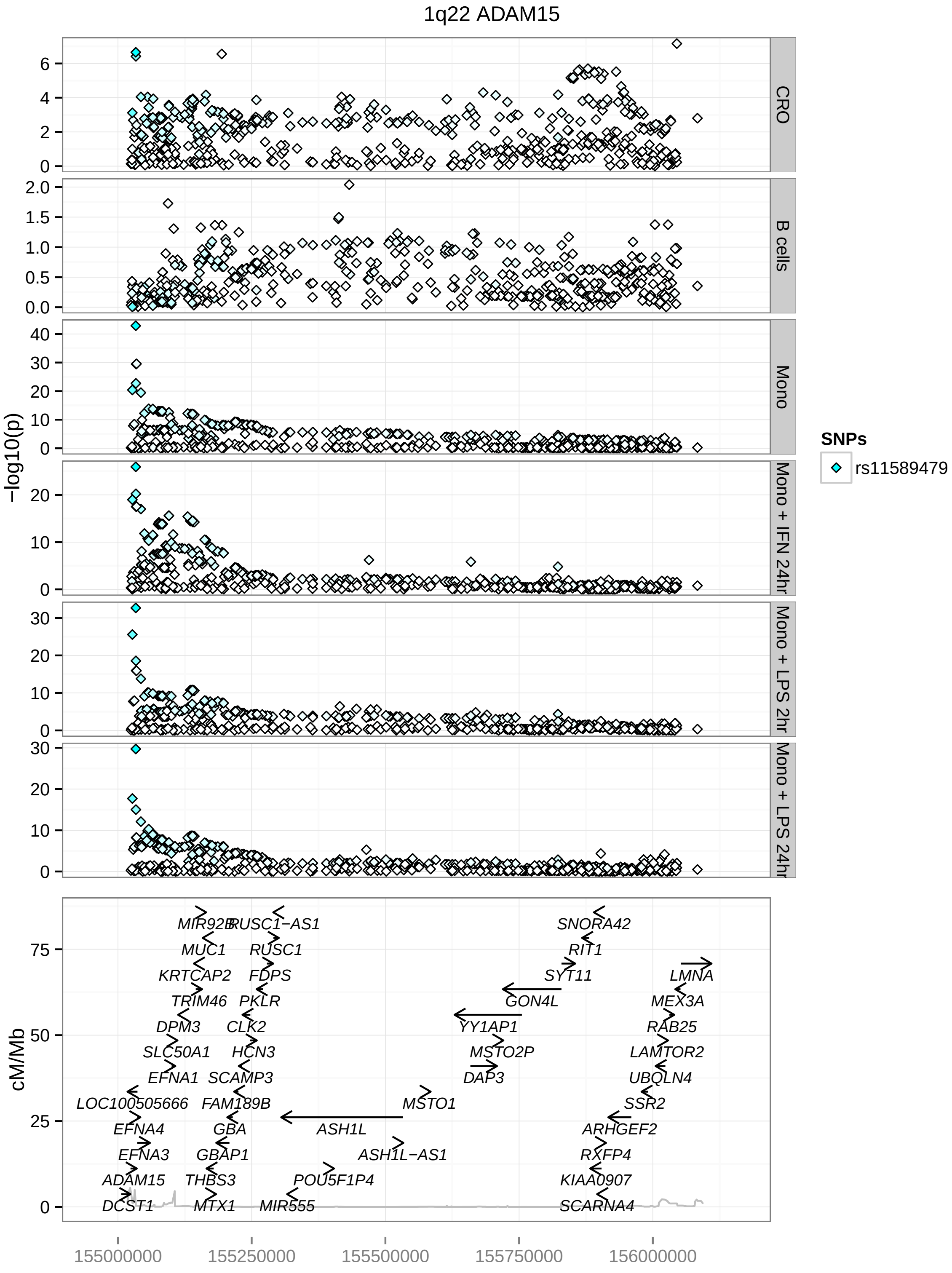

Supplementary Figure 1. Chromosome 1q22 expression of *ADAM15* (probe ILMN_1751500)

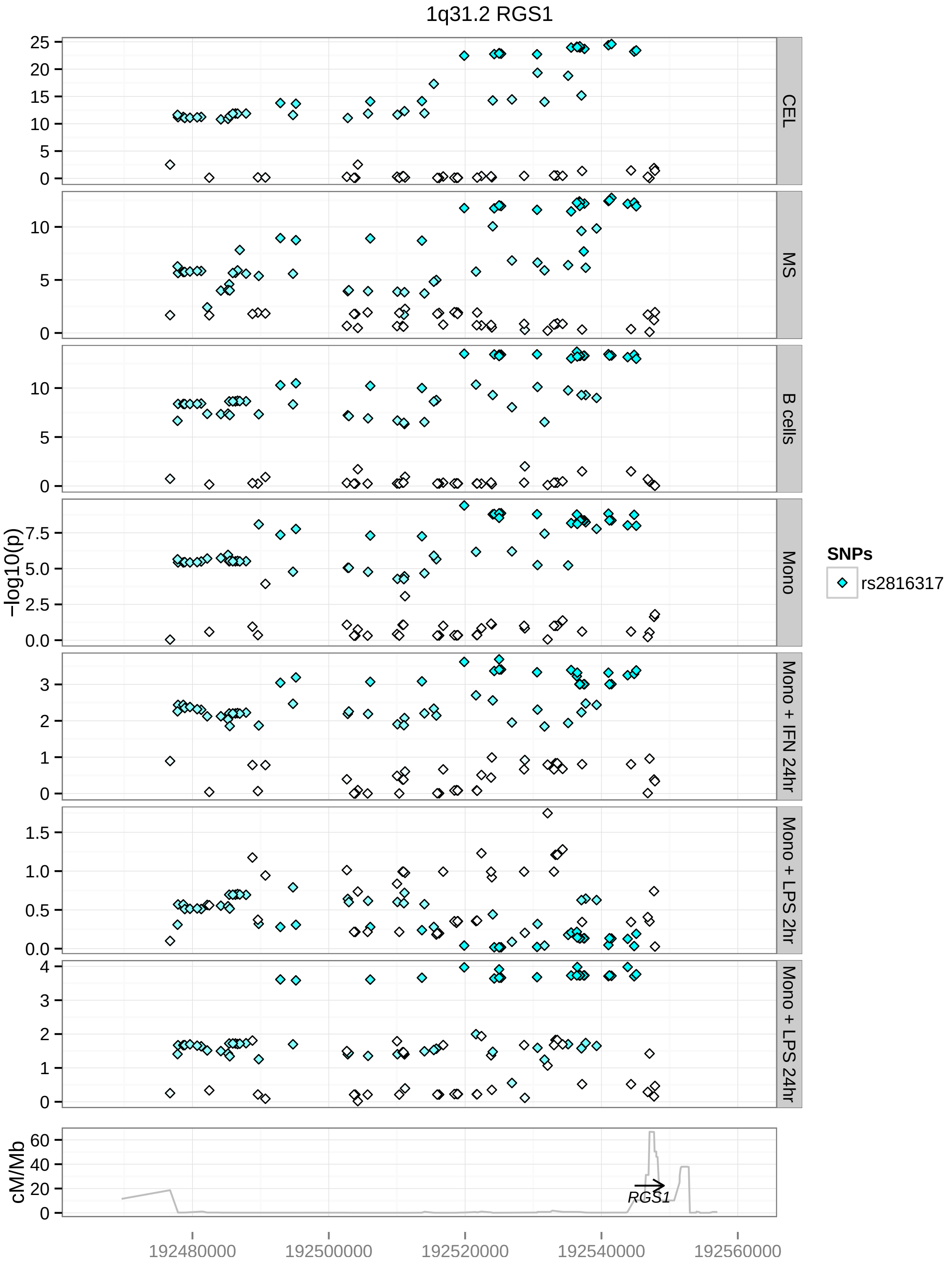

Supplementary Figure 2. Chromosome 1q31.2 expression of *RGS1* (probe ILMN_1656011)

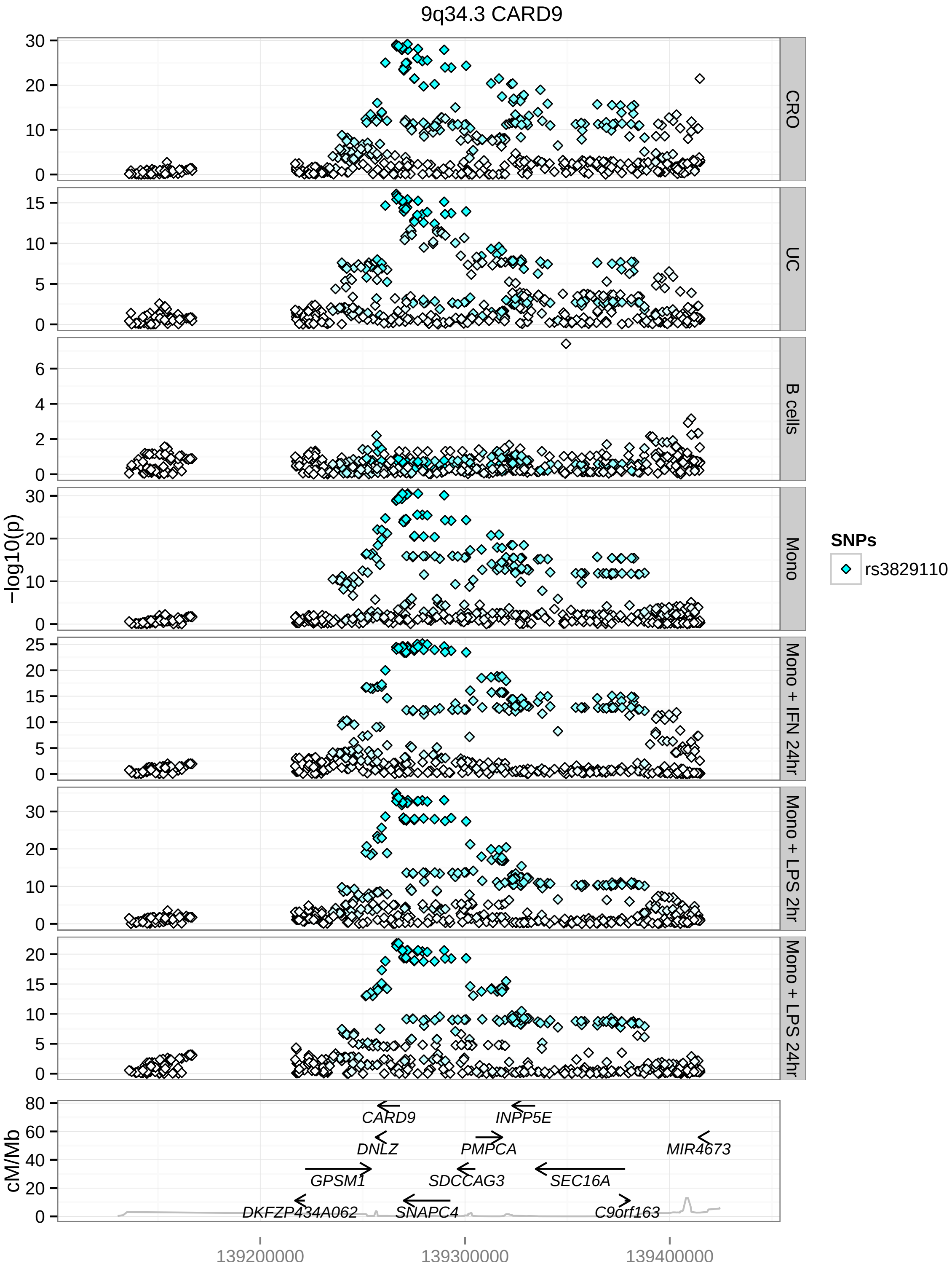

Supplementary Figure 3. Chromosome 9q34.3 expression of *CARD9* (probe ILMN_1712532)

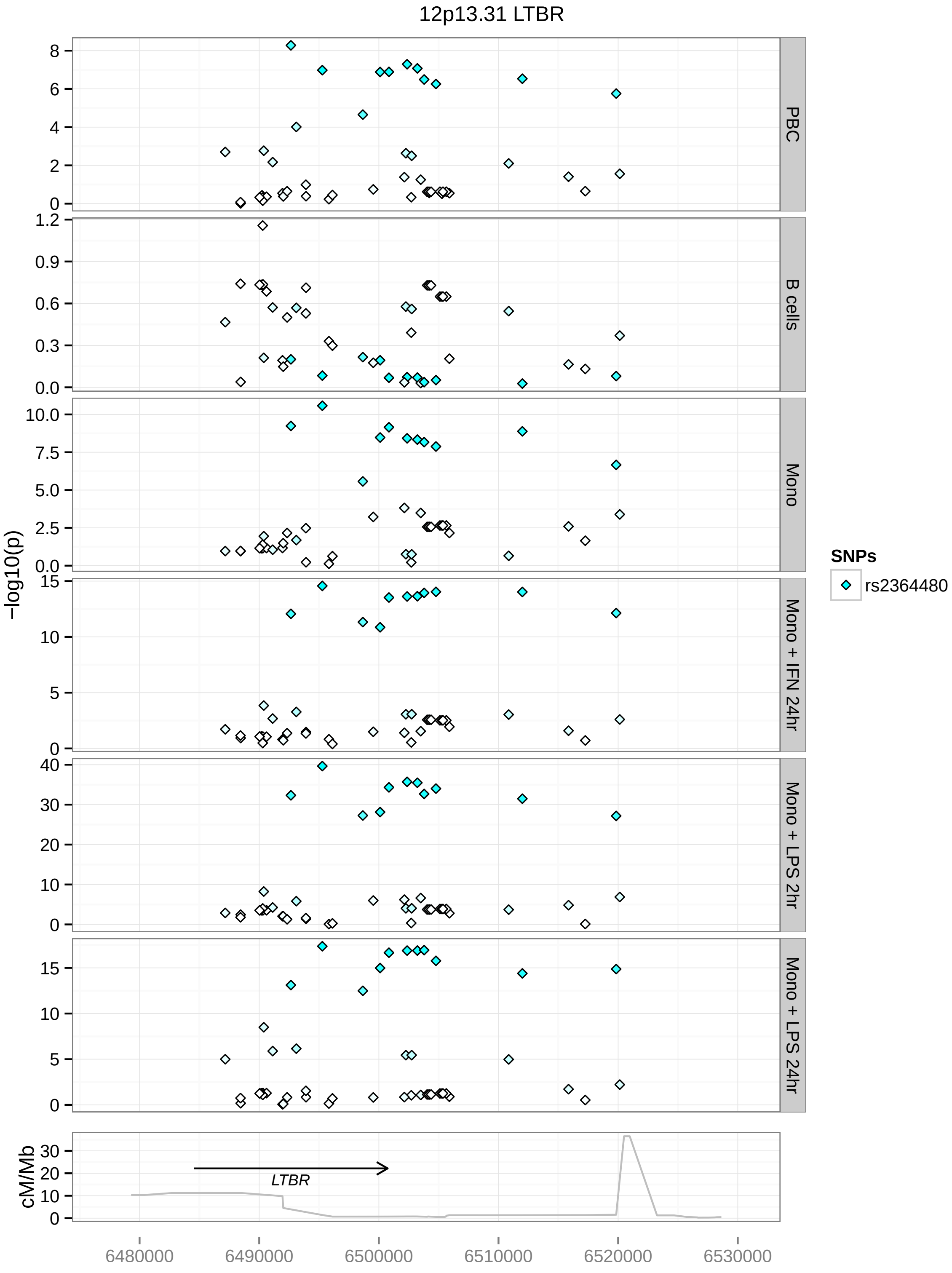

Supplementary Figure 4. Chromosome 12p13.31 expression of *LTBR* (probe ILMN_1667476)

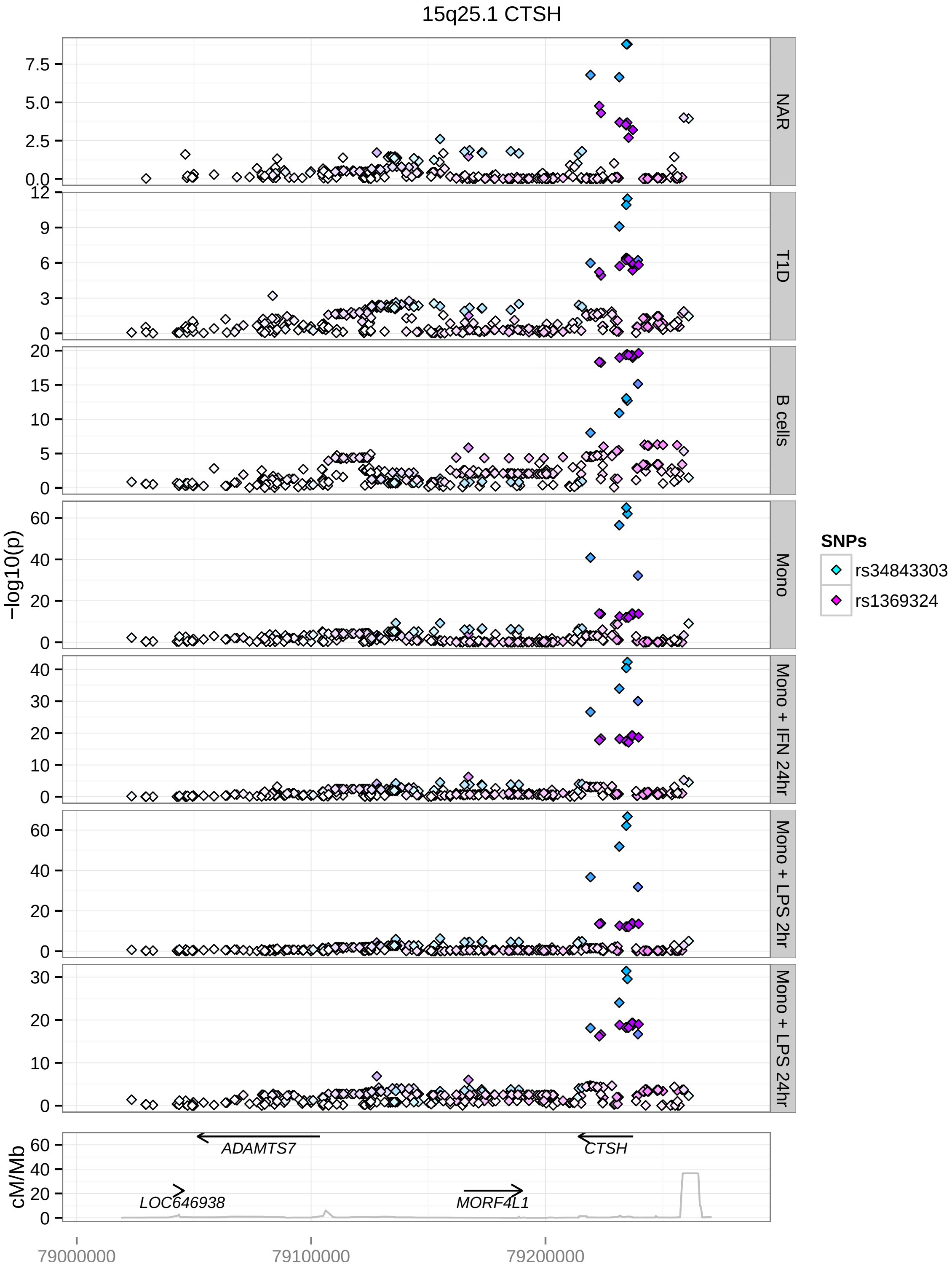

Supplementary Figure 5. Chromosome 15q25.1 expression of *CTSH* (probe ILMN_2390853)

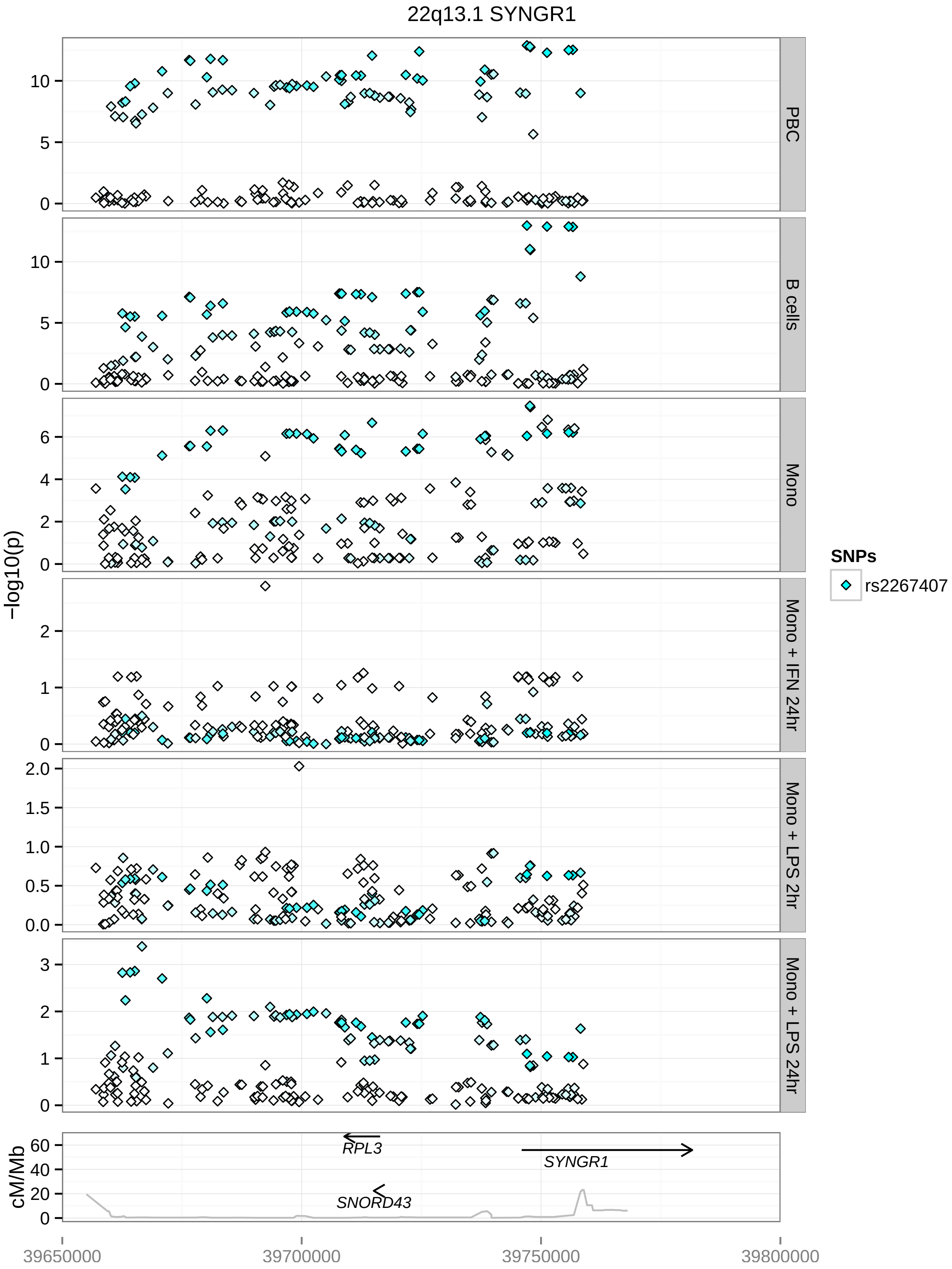

Supplementary Figure 6. Chromosome 22q13.1 expression of *SYNGR1* (probe ILMN_1727805)

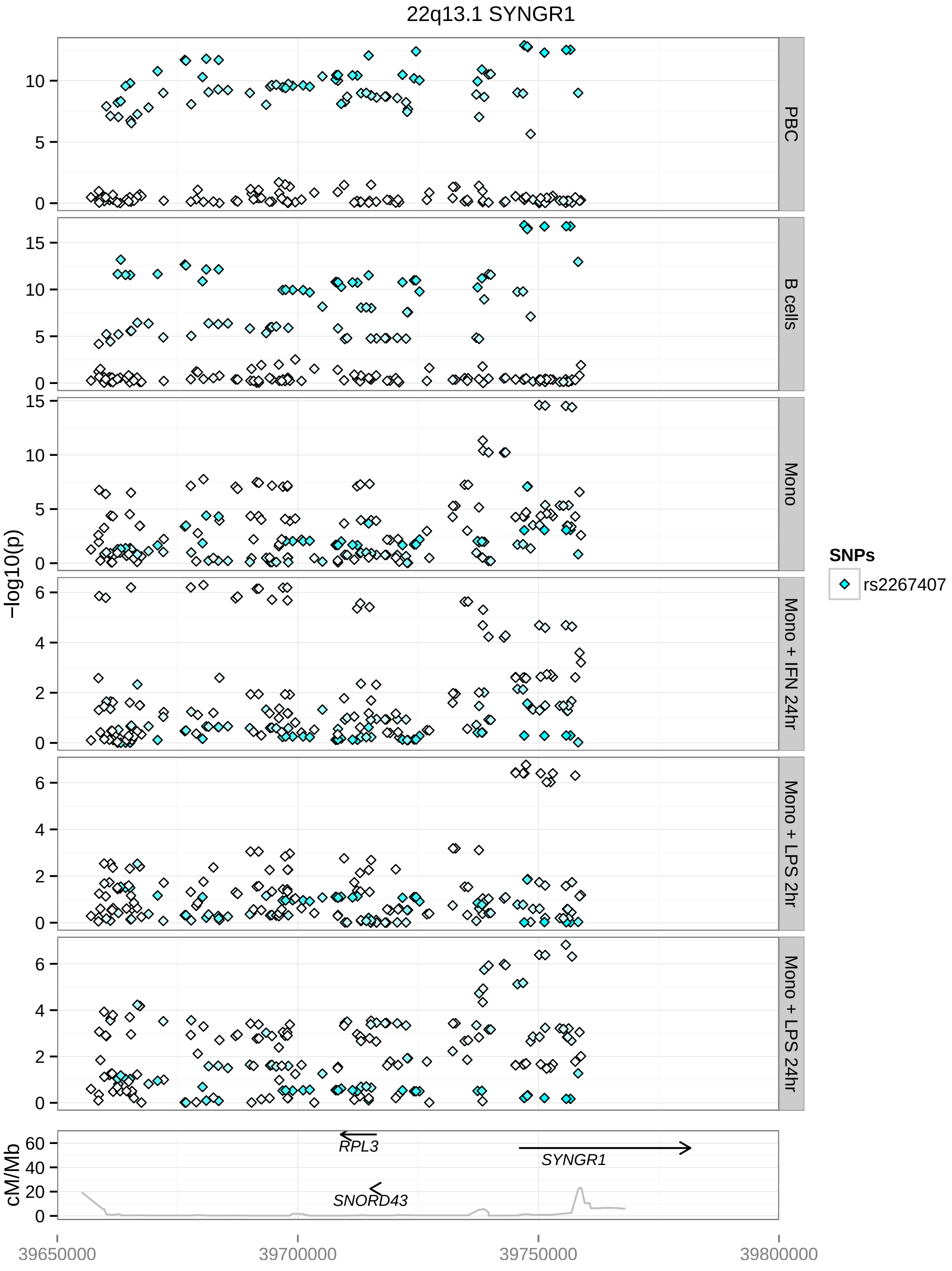

Supplementary Figure 7. Chromosome 22q13.1 expression of *SYNGR1* (probe ILMN_1810875)

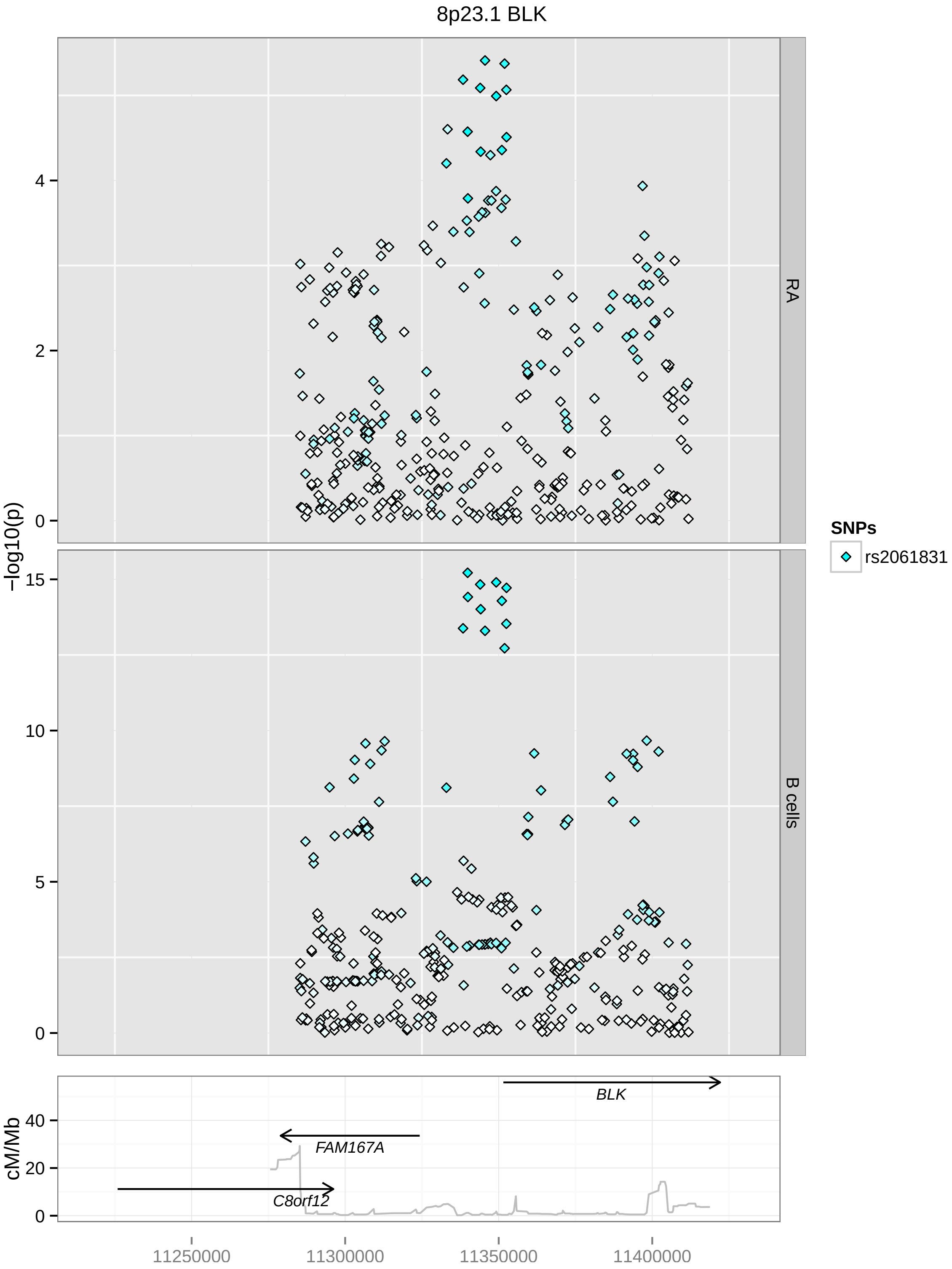

Supplementary Figure 8. Chromosome 8p23.1 expression of *BLK* (probe ILMN_1668277)

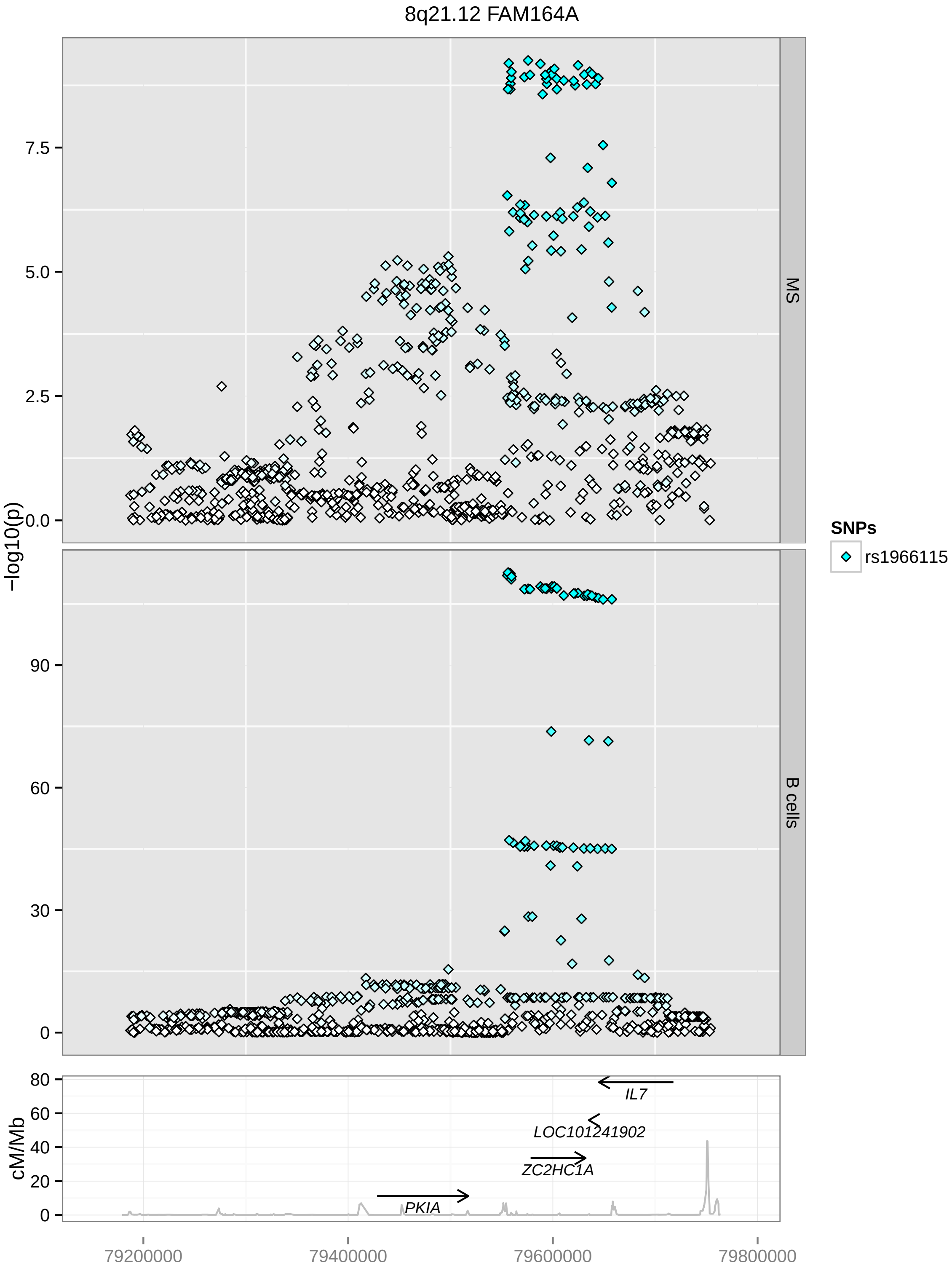

Supplementary Figure 9. Chromosome 8q21.12 expression of *FAM164A* (probe ILMN_1789558)

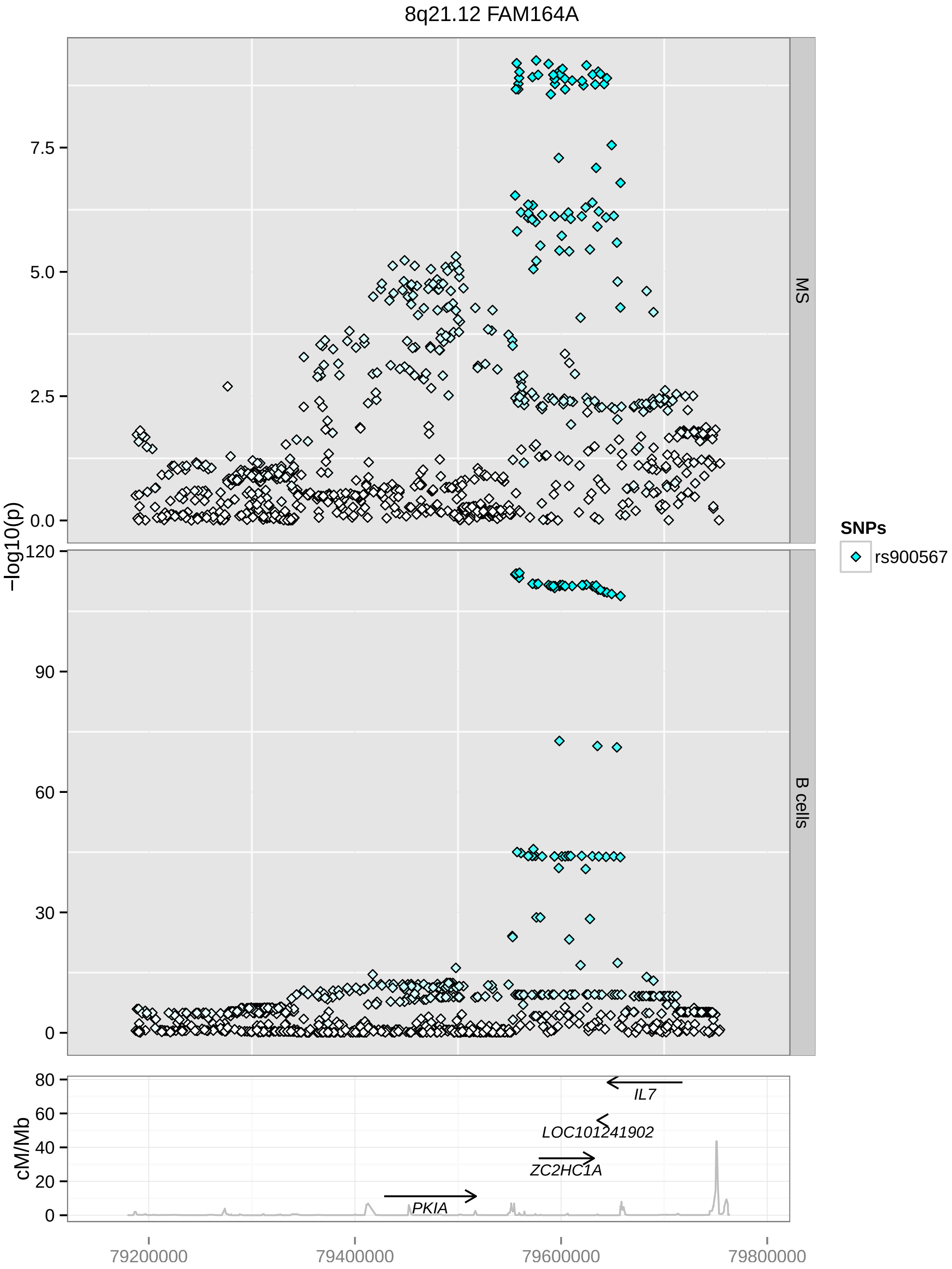

Supplementary Figure 10. Chromosome 8q21.12 expression of *FAM164A* (probe ILMN_2057981)

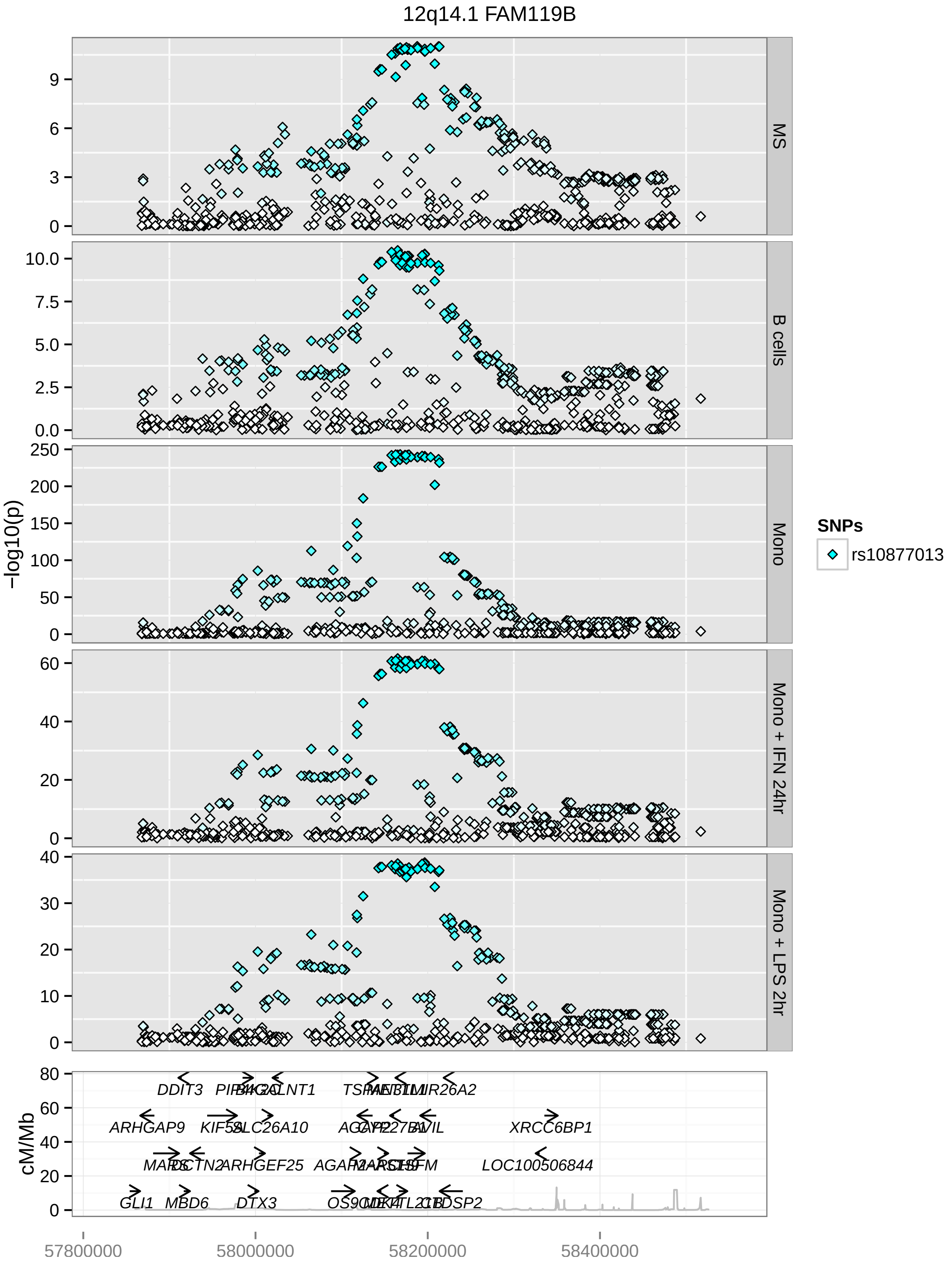

Supplementary Figure 11. Chromosome 12q14.1 expression of *FAM119B* (probe ILMN_1723846)

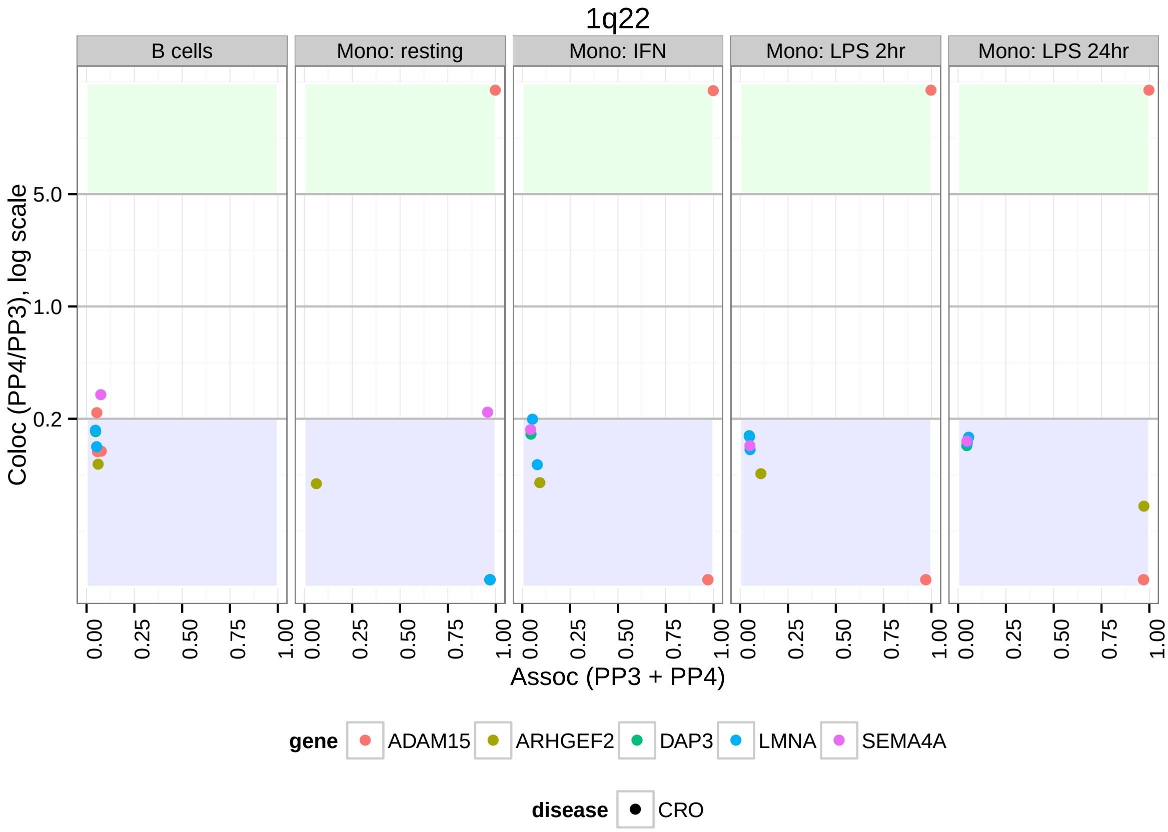

Supplementary Figure 12. Evidence for colocalisation across chromosome 1q22 region. This and each plot from Supplementary Figures 13–20 shows the posterior support for association of both gene expression and disease to a region ($PP_3 + PP_4$, x axis) and odds for colocalisation ($PP_4 : PP_3$, y axis, log scale) across all cell types considered and all genes with evidence of an eQTL in at least one cell type. Colocalising signals are located in the top right of any plot. Where multiple probes exist for a gene, they are shown by the same symbol, allowing us to highlight regions where colocalisation is specific to a single probe (isoform-specific colocalisation). In no case do we find multiple genes colocalising with a disease, or the same gene colocalising across all cells considered. In most cases, the colocalisation is disease-specific. Green (blue) shading is used to indicate regions where the odds PP4:PP3 $> 5 : 1$ ($< 1 : 5$), indicating colocalising (distinct) effects, but note that interpretion also depends on the evidence for assocation (PP3 + PP4, x axis).

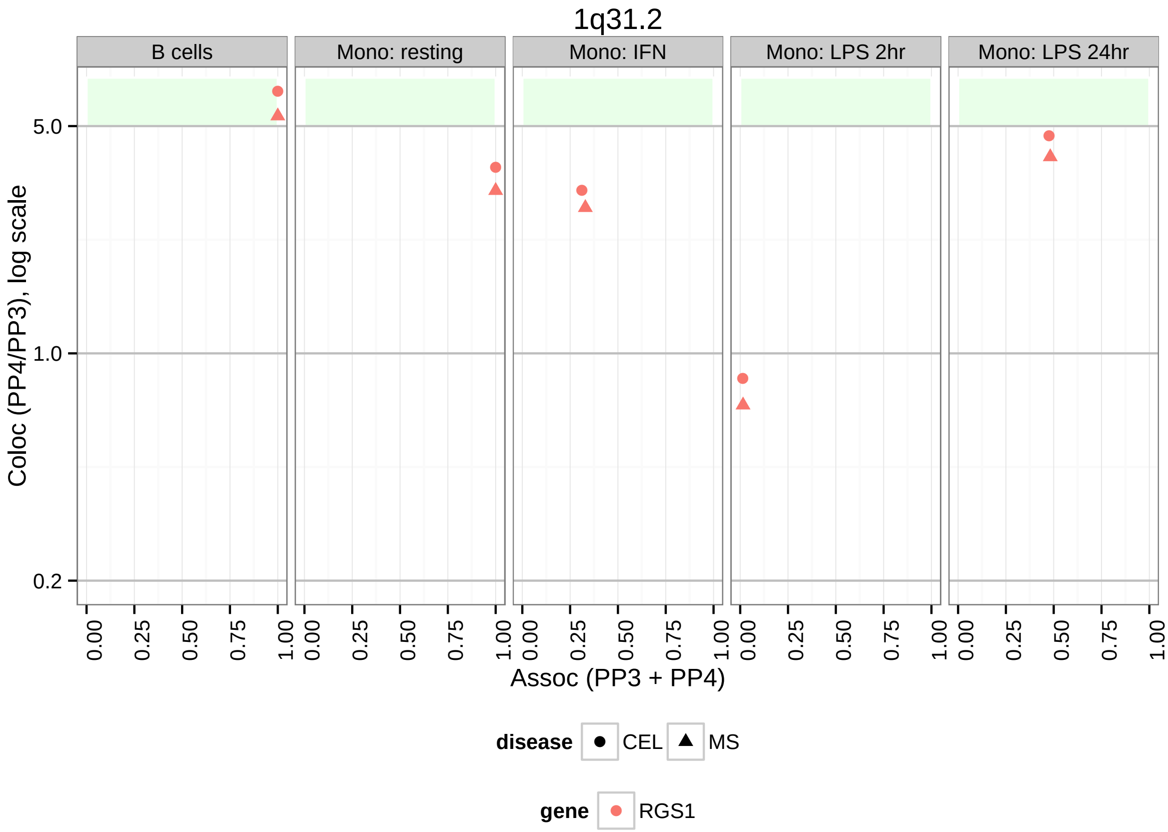

Supplementary Figure 13. Evidence for colocalisation across chromosome 1q31.2 region. See Supplementary Figure 12 for details.

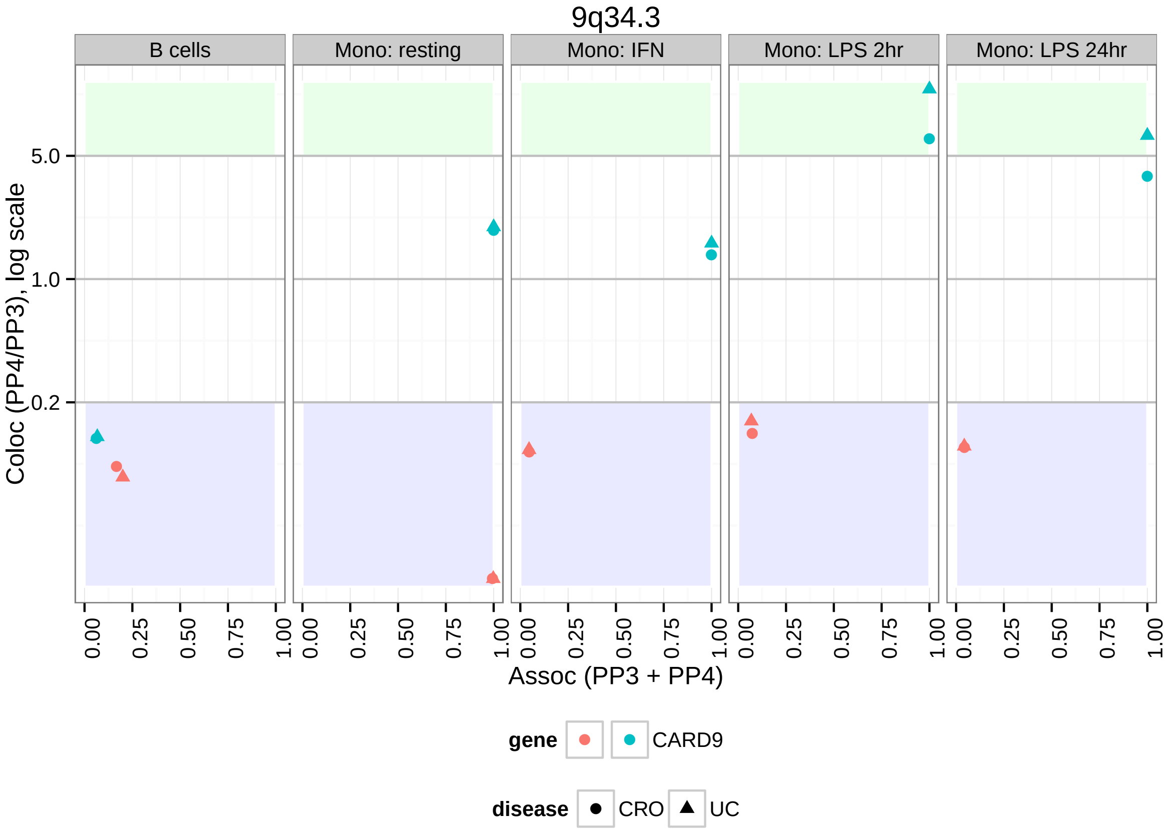

Supplementary Figure 14. Evidence for colocalisation across chromosome 9q34.3 region. See Supplementary Figure 12 for details.

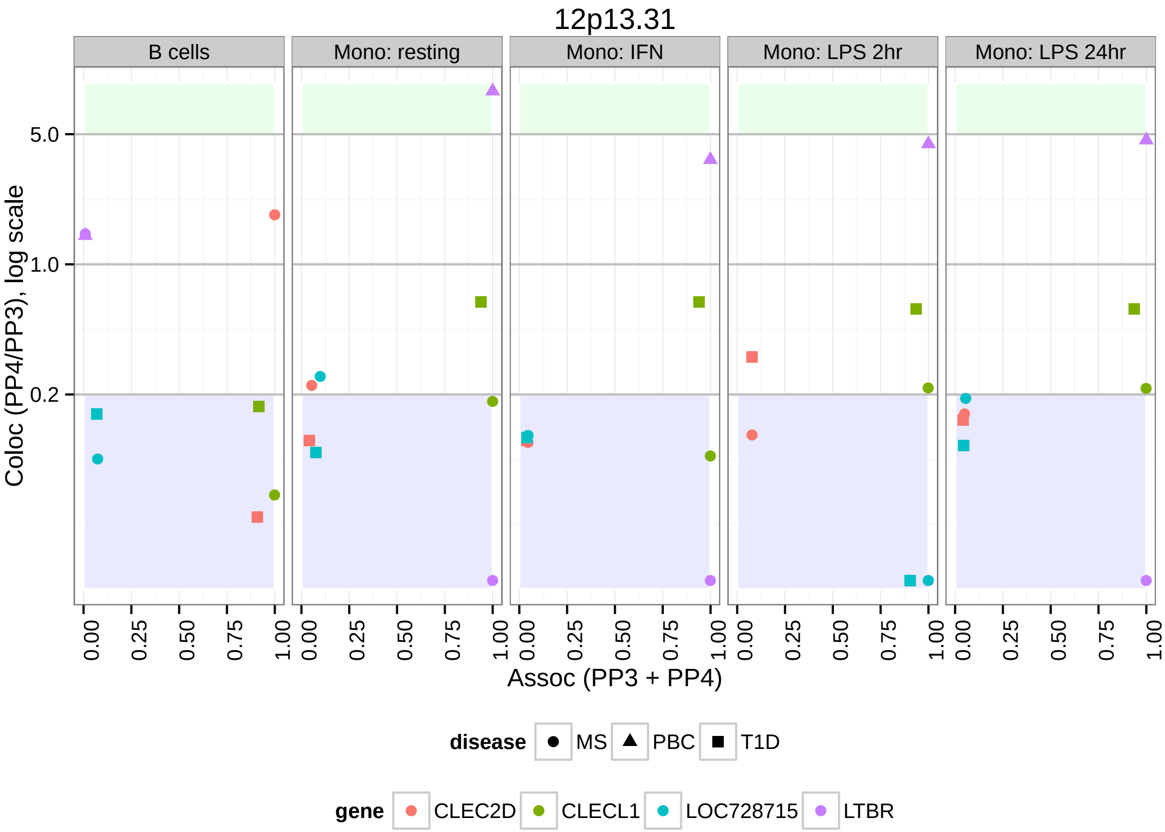

Supplementary Figure 15. Evidence for colocalisation across chromosome 12p13.31 region. See Supplementary Figure 12 for details.

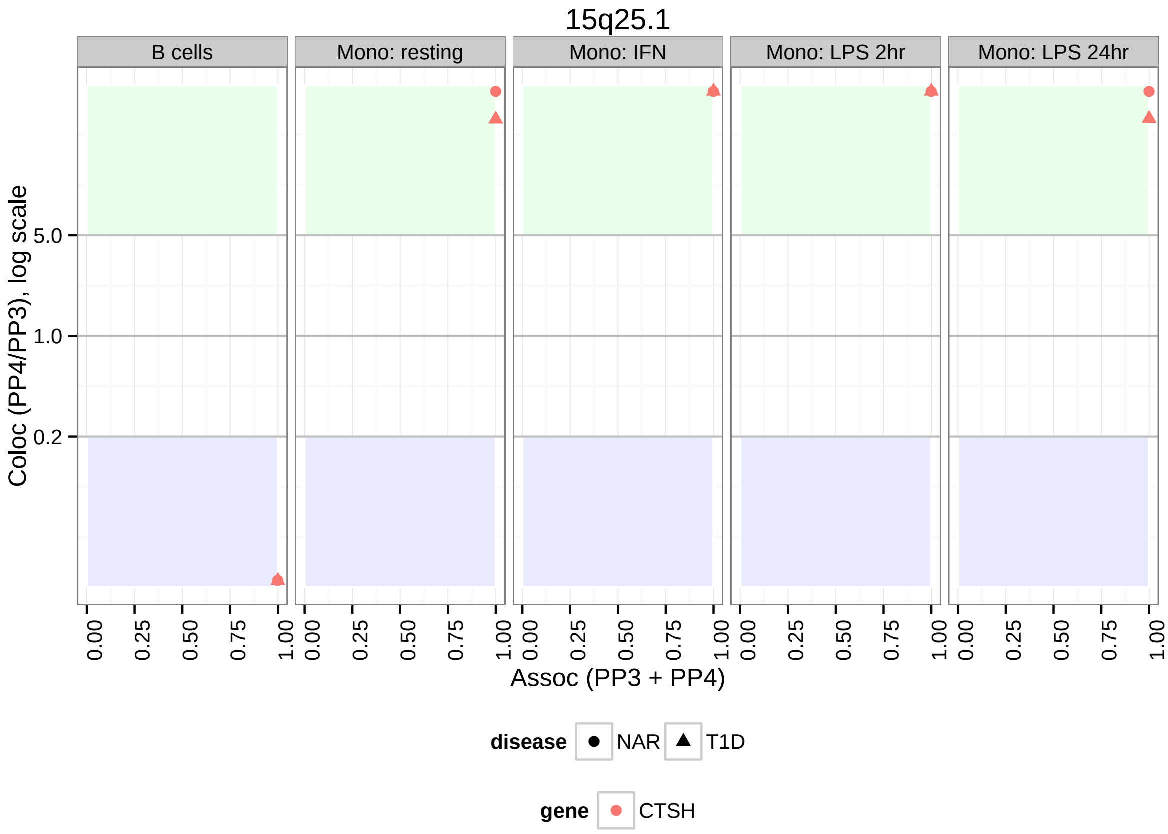

Supplementary Figure 16. Evidence for colocalisation across chromosome 15q25.1 region. See Supplementary Figure 12 for details.

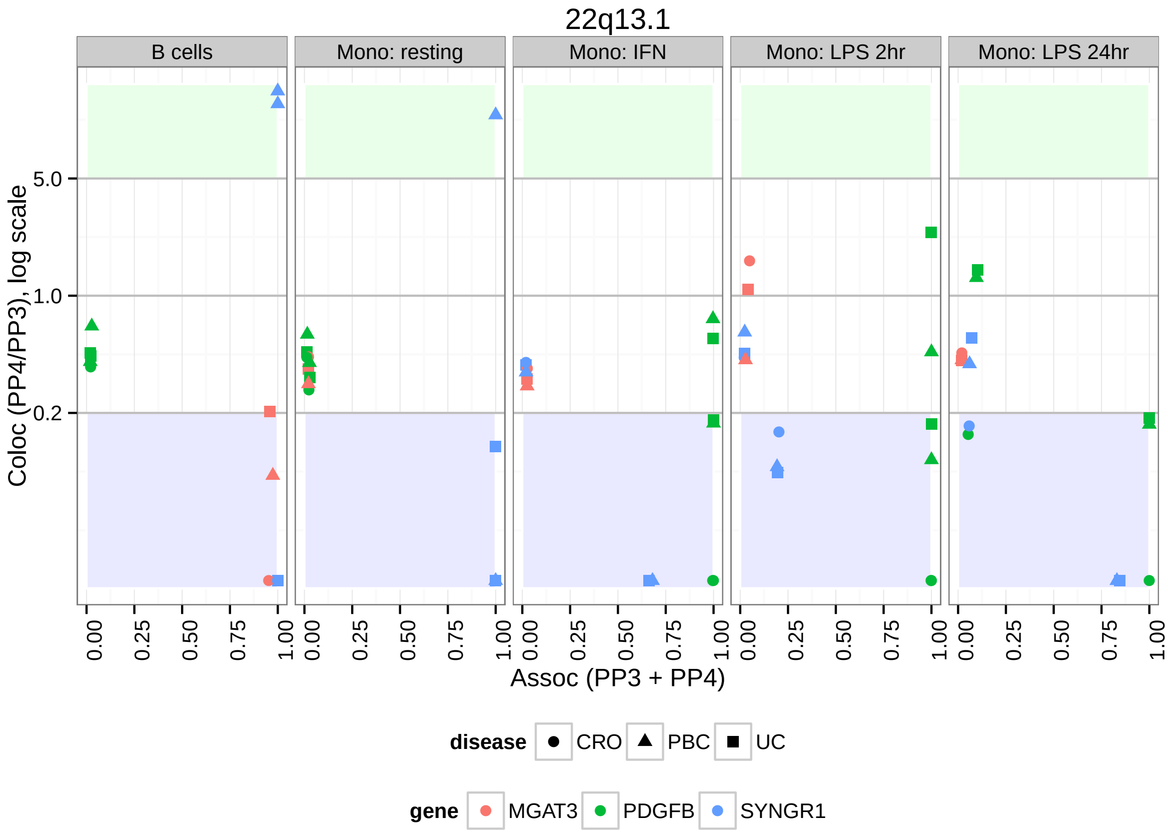

Supplementary Figure 17. Evidence for colocalisation across chromosome 22q13.1 region. See Supplementary Figure 12 for details.

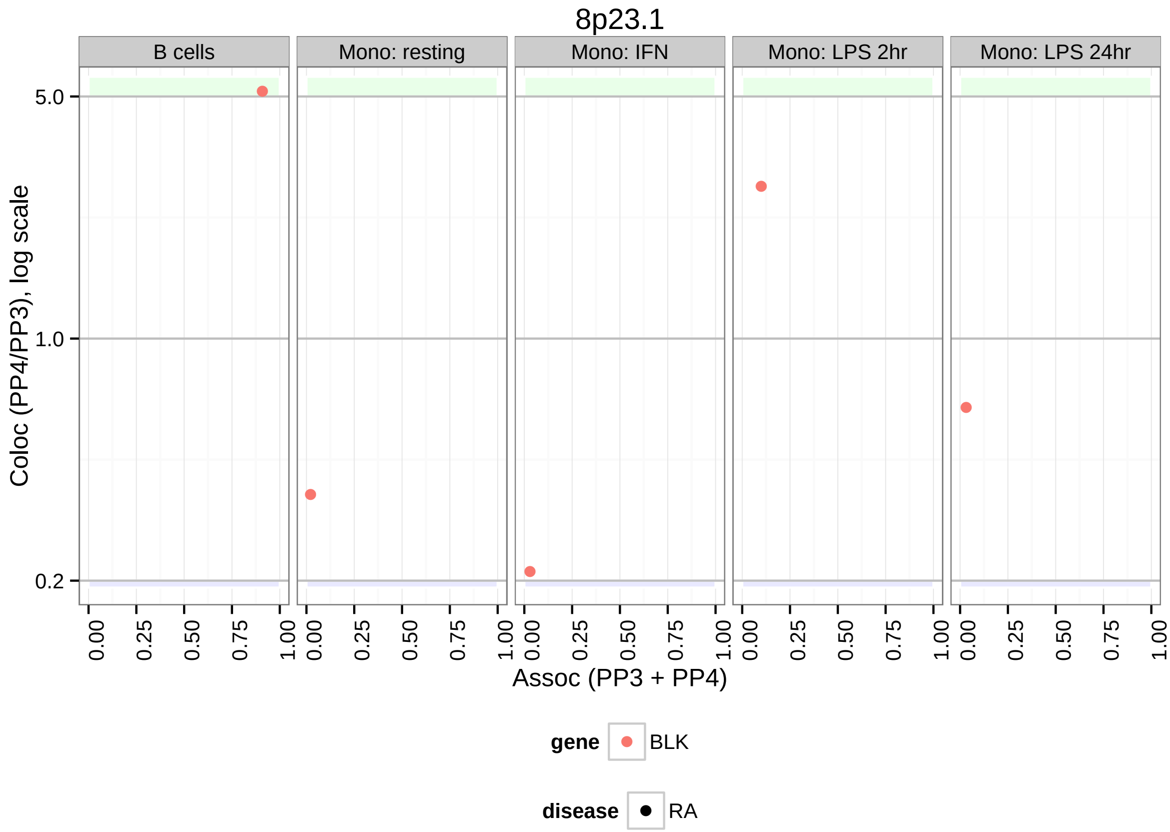

Supplementary Figure 18. Evidence for colocalisation across chromosome 8p23.1 region. See Supplementary Figure 12 for details.

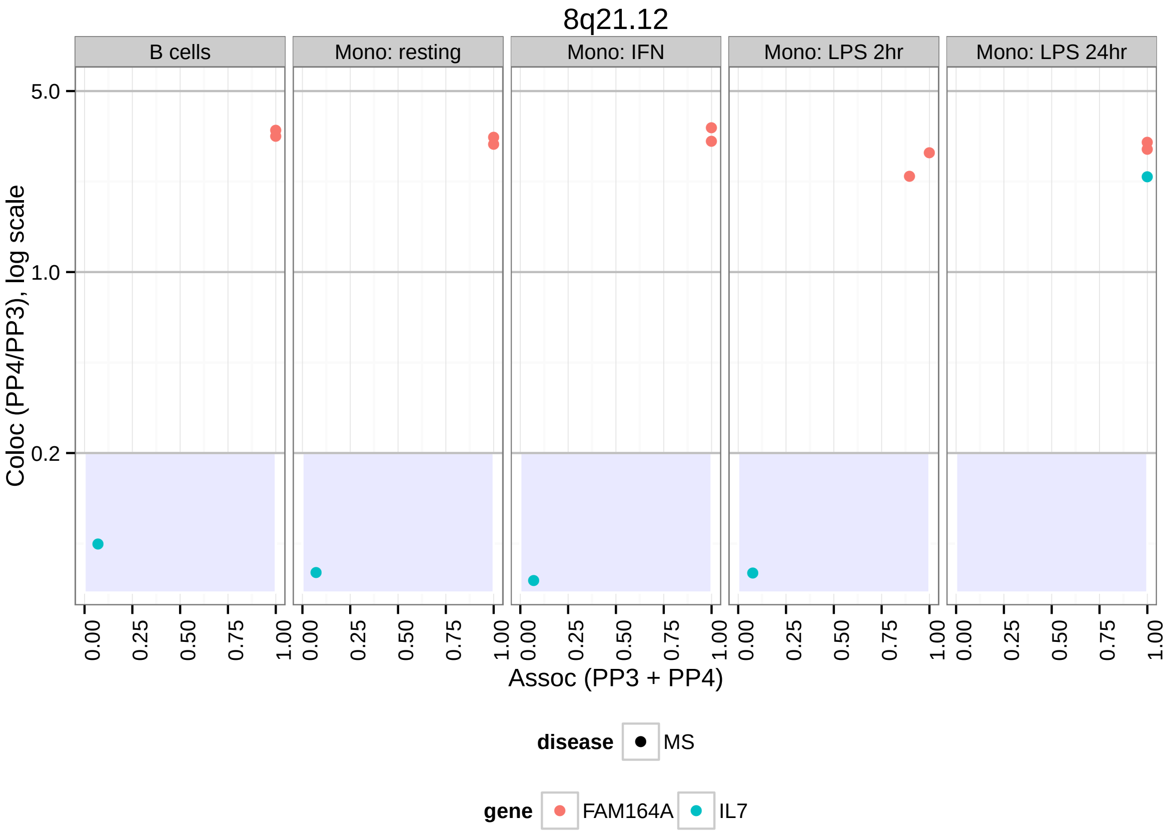

Supplementary Figure 19. Evidence for colocalisation across chromosome 8q21.12 region. See Supplementary Figure 12 for details.

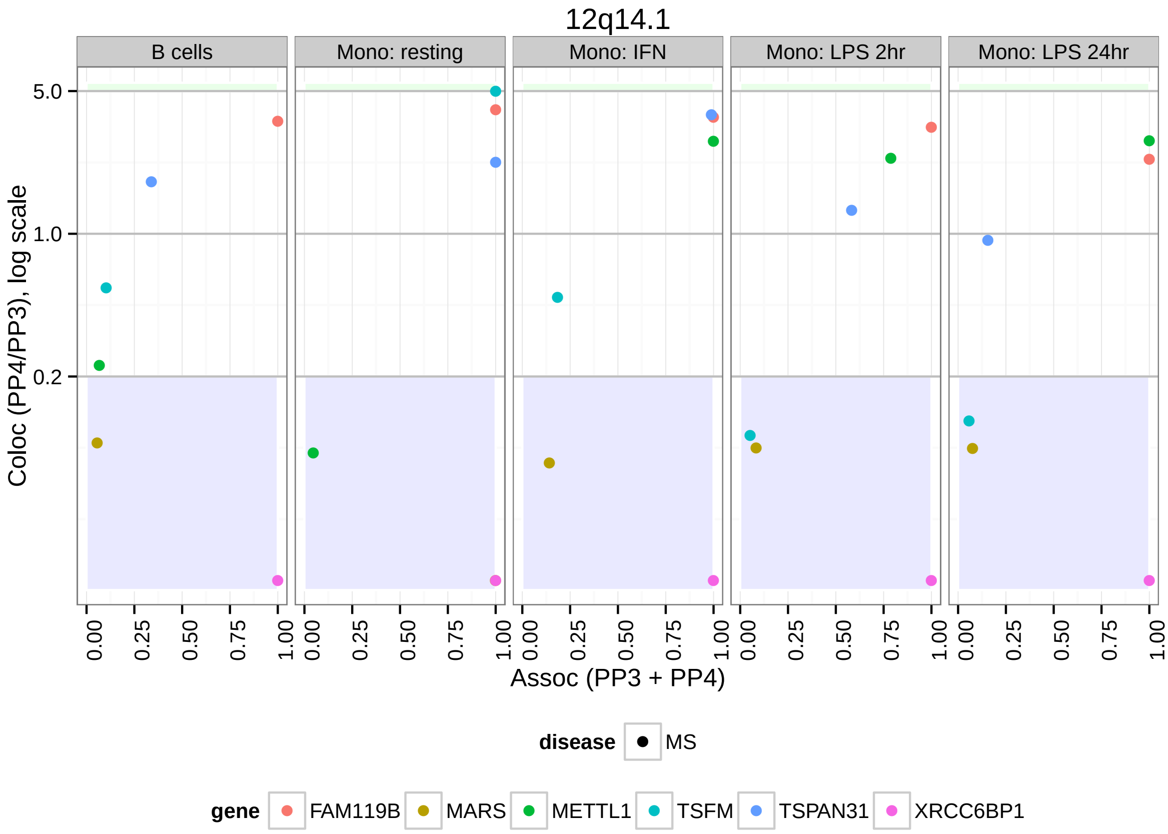

Supplementary Figure 20. Evidence for colocalisation across chromosome 12q14.1 region. See Supplementary Figure 12 for details.

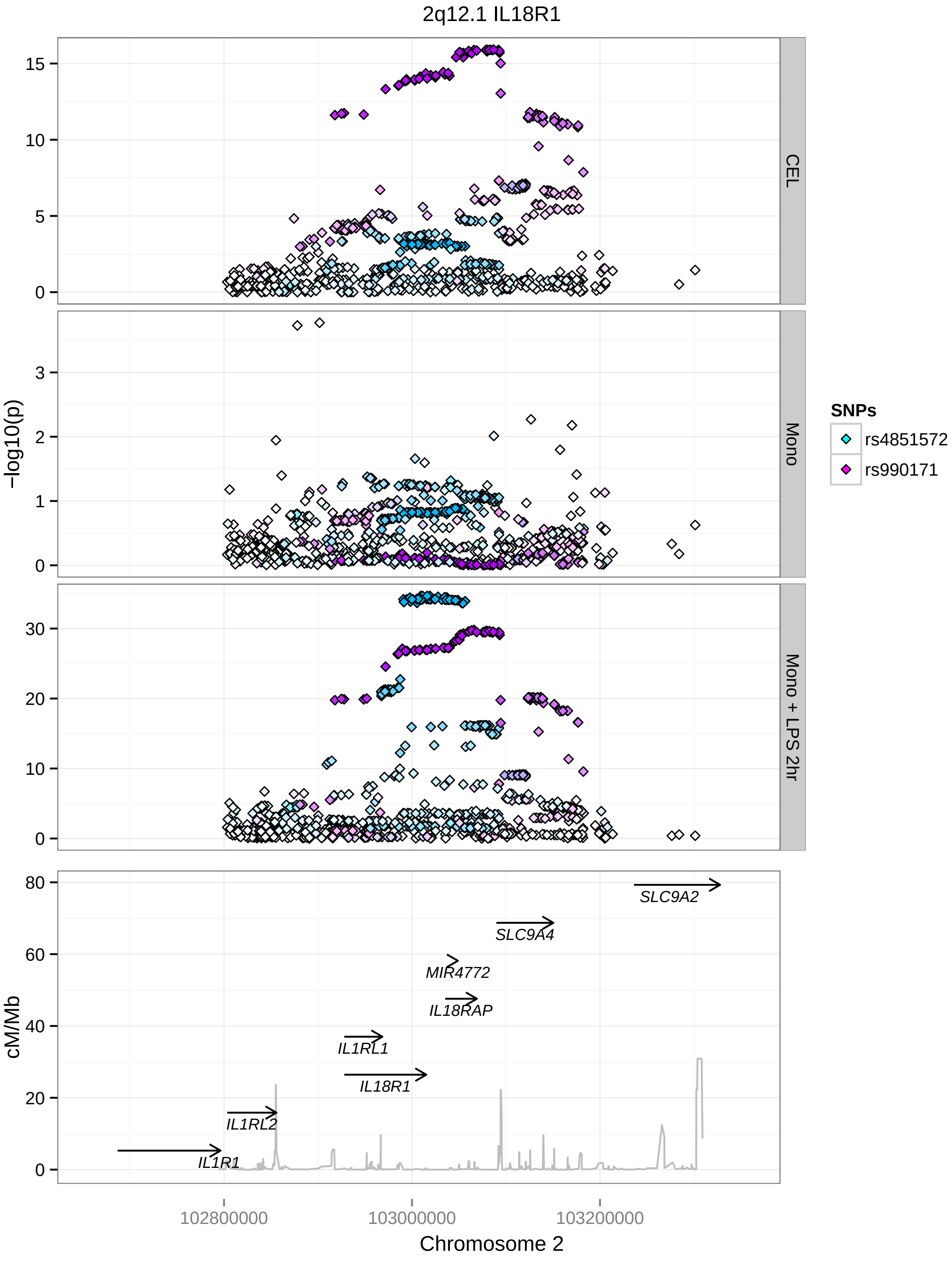

Supplementary Figure 21. Chromosome 2q12.1 expression of *IL18R1* (probe ILMN_1781700)

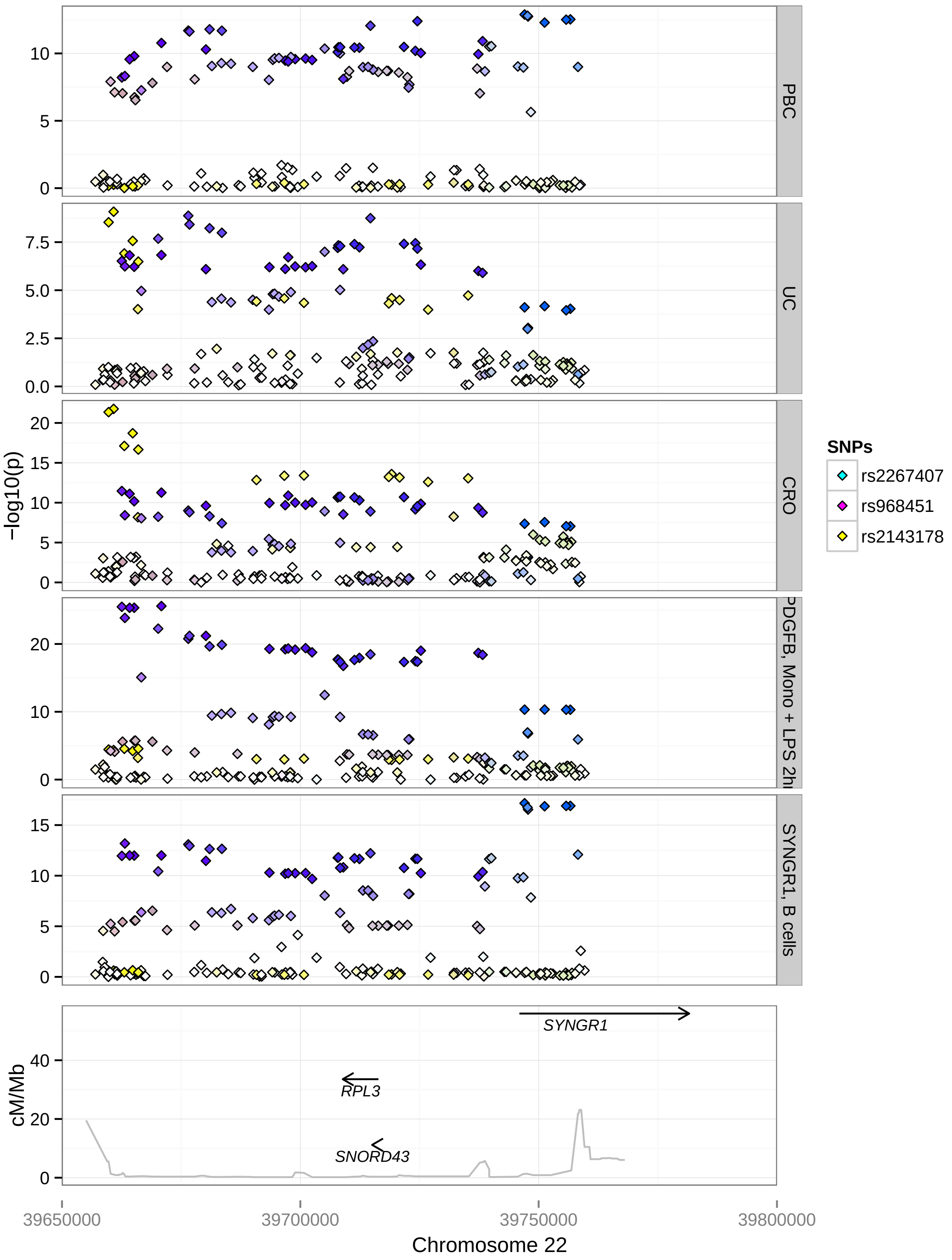

Supplementary Figure 22. Chromosome 22q13.1 expression of *PDGFB* (probe ILMN_1775822) in monocytes stimulated with LPS for 2 hours and *SYNGR1* (probe ILMN_1810875) in B cells. *PDGFB* is located 160kb outside the region shown, at chr22:39,619,364..39,640,957

Supplementary Table 1. Complete results for regions showing evidence for association with both gene expression and disease ($PP_4 + PP_3 \geq 0.8$). - gene expression (unstimulated monocytes and B cells, Interferon-γ and Lipopolysaccharide stimulated monocytes) and autoimmune diseases. PP_4 and PP_3 are posterior probabilities of shared causal variant and of distinct causal variants. IFN24 = Monocytes, after 24 hours interferon-γ stimulation; LPS2 = Monocytes, after 2 hours lipopolysaccharide stimulation; LPS24: = Monocytes, after 24 hours lipopolysaccharide stimulation.

Region	Gene	Probe	SNPs	B cells		Monocytes		IFN24		LPS2		LPS24		Disease
				Assoc	Coloc	Assoc	Coloc	Assoc	Coloc	Assoc	Coloc	Assoc	Coloc	
1p36.32	*HES5*	ILMN_1794742	289	0.02	0.28	0.04	0.17	0.85	0.01	0.02	0.34	0.02	0.27	ATD
			272	0.03	0.28	0.04	0.17	0.99	0	0.03	0.43	0.02	0.25	CEL
			366	0.03	0.21	0.05	0.13	1	0	0.04	0.28	0.03	0.19	MS
			249	0.02	0.29	0.04	0.24	0.96	0.02	0.03	0.35	0.02	0.3	RA
			360	0.03	0.21	0.04	0.15	0.8	0.01	0.03	0.33	0.02	0.23	UC
	MMEL1	ILMN_1718488	289	0.04	0.14	0.93	1.2	0.92	1.51	0.52	1.77	0.04	0.46	ATD
			272	0.04	0.17	1	0.05	0.98	2.34	0.57	2.86	0.04	0.46	CEL
			366	0.05	0.12	1	0.3	0.98	1.59	0.62	2.42	0.05	0.38	MS
			249	0.03	0.21	0.99	2.79	0.96	1.68	0.43	1	0.04	0.6	RA
			360	0.04	0.13	0.83	0.24	0.88	1.13	0.45	1.27	0.04	0.3	UC
1p36.23	*UTS2*	ILMN_1671818	455	0.86	0	0.86	0	0.86	0	0.86	0	0.86	0	CRO
			688	1	0	1	0	1	0	1	0	1	0	UC
		ILMN_1732198	455	0.86	0	0.86	0	0.86	0	0.86	0	0.86	0	CRO
			688	1	0	1	0	1	0	1	0	1	0	UC
		ILMN_1775780	455	0.04	0.21	0.86	0	0.74	0	0.86	0	0.86	0	CRO
			688	0.07	0.16	1	0	0.94	0	1	0	1	0	UC
		ILMN_2236625	455	0.83	0	0.86	0	0.86	0	0.86	0	0.86	0	CRO
			688	1	0	1	0	1	0	1	0	1	0	UC
	ERRFI1	ILMN_1665510	688	0.1	0.1	0.05	0.38	1	0	1	0	0.99	0	UC
	PARK7	ILMN_1744713	688	0.09	0.11	1	0.19	1	0.17	0.86	0.08	0.71	0.2	UC
			359	0.03	0.2	0.83	2.31	0.75	0.99	0.68	2.26	0.68	2.06	CRO
1p36.13	*PADI4*	ILMN_1807529	202	0.02	0.41	1	0	0.05	0.89	0.03	0.5	1	0	RA
			201	0.02	0.4	1	0	0.06	1.05	0.03	0.51	1	0	RA
1p22.1	*RPAP2*	ILMN_1657754	1117	0.9	0	1	0	1	0	0.65	0	1	0	MS
1p13.2	*AP4B1*	ILMN_1669377	643	0.99	0	0.2	1.34	1	0	1	0	1	0	RA
			671	0.99	0	0.2	1.3	1	0	1	0	1	0	T1D
			576	0.99	0	0.15	0.87	1	0	1	0	1	0	ATD
			677	0.99	0	0.17	0.86	1	0	1	0	1	0	CRO
	PHTF1	ILMN_1803464	643	0.08	0.09	1	0	1	0	1	0	1	0	RA
			671	0.08	0.09	1	0	1	0	1	0	1	0	T1D
			576	0.08	0.1	1	0	1	0	1	0	1	0	ATD
			677	0.09	0.09	1	0	1	0	1	0	1	0	CRO
	PTPN22	ILMN_1695640	643	0.97	0.01	0.05	0.21	1	0	0.06	0.14	0.06	0.19	RA
			671	0.97	0.01	0.06	0.2	1	0	0.06	0.13	0.06	0.18	T1D

Continued on next page

Supplementary Table 1 – continued from previous page

Region	Gene	Probe	SNPs	B cells		Monocytes		IFN24		LPS2		LPS24		Disease
				Assoc	Coloc	Assoc	Coloc	Assoc	Coloc	Assoc	Coloc	Assoc	Coloc	
			576	0.97	0	0.05	0.23	1	0	0.05	0.16	0.05	0.24	ATD
			677	0.97	0	0.06	0.2	1	0	0.06	0.13	0.06	0.16	CRO
		ILMN_1715885	643	1	0	0.52	0.22	1	0	0.06	0.13	0.06	0.23	RA
			671	1	0	0.54	0.23	1	0	0.07	0.13	0.06	0.22	T1D
			576	1	0	0.52	0.18	1	0	0.06	0.16	0.05	0.25	ATD
			677	1	0	0.51	0.21	1	0	0.07	0.12	0.06	0.17	CRO
		ILMN_2246328	643	1	0	1	0	1	0	0.53	0.02	0.53	0.02	RA
			671	1	0	1	0	1	0	0.53	0.02	0.53	0.02	T1D
			576	1	0	1	0	1	0	0.53	0.03	0.54	0.02	ATD
			677	1	0	1	0	1	0	0.54	0.02	0.53	0.01	CRO
1q22	*ADAM15*	ILMN_1751500	523	0.05	0.22	**1**	**22.16**	**1**	**22.06**	**1**	**22.16**	**1**	**22.16**	CRO
		ILMN_1756920	523	0.06	0.12	0.97	0	0.97	0	0.97	0	0.97	0	CRO
		ILMN_2384544	523	0.08	0.13	0.97	0	0.97	0	0.97	0	0.97	0	CRO
	ARHGEF2	ILMN_1703477	523	0.06	0.1	0.06	0.08	0.09	0.08	0.11	0.09	0.97	0.06	CRO
1q23.3	*FCGR2B*	ILMN_1660027	218	0.06	0.1	1	0	1	0	0.03	0.24	0.93	0	UC
			159	0.05	0.22	0.99	0	1	0	0.02	0.36	0.46	0.01	CRO
		ILMN_2382403	218	0.04	0.38	1	0	1	0	0.04	0.23	0.91	0	UC
			159	0.03	0.36	1	0	1	0	0.03	0.31	0.45	0.01	CRO
	FCRLB	ILMN_1782015	218	0.1	0.7	1	0	1	0	0.04	0.37	0.48	0.02	UC
	HSPA6	ILMN_1806165	218	1	0	1	0	0.9	0	0.13	0.09	0.02	0.34	UC
			159	1	0	1	0	0.93	0	0.13	0.05	0.02	0.55	CRO
	SDHC	ILMN_2323366	218	0.04	0.21	0.78	0	0.97	0	0.03	0.21	0.09	0.26	UC
1q31.2	*RGS1*	ILMN_1656011	83	**1**	**6.41**	**1**	**3.74**	0.31	3.17	0.01	0.84	0.48	4.67	CEL
			101	**1**	**5.37**	**1**	**3.17**	0.33	2.81	0.01	0.69	0.48	4.02	MS
1q31.3	*C1orf53*	ILMN_1661595	597	0.05	0.12	1	0	1	0	0.07	0.11	0.8	0	CRO
			580	0.05	0.12	1	0	1	0	0.07	0.11	0.53	0.01	PBC
			593	0.05	0.12	0.94	0	0.94	0	0.08	0.09	0.8	0	UC
1q32.1	*IL19*	ILMN_1682592	363	0.04	0.29	0.06	0.11	1	0	1	0	0.21	0.03	UC
			240	0.03	0.58	0.03	0.24	1	0	1	0	0.19	0.04	CRO
			236	0.03	0.52	0.02	0.28	1	0	1	0	0.2	0.05	T1D
		ILMN_1799575	363	0.09	0.09	0.03	0.28	1	0	0.99	0	0.13	0.06	UC
			240	0.04	0.28	0.02	0.41	1	0	0.99	0	0.12	0.06	CRO
			236	0.04	0.3	0.02	0.38	1	0	0.98	0	0.14	0.08	T1D
	IL24	ILMN_1774685	363	0.06	0.14	0.03	0.23	1	0	0.04	0.23	0.11	0.06	UC
			240	0.05	0.17	0.02	0.37	1	0	0.03	0.34	0.16	0.04	CRO
			236	0.06	0.17	0.02	0.37	1	0	0.03	0.33	0.17	0.18	T1D
		ILMN_2407799	363	0.04	0.26	0.05	0.25	1	0	0.07	0.12	0.08	0.1	UC
			240	0.03	0.37	0.03	0.36	1	0	0.04	0.22	0.1	0.07	CRO
			236	0.03	0.36	0.03	0.47	1	0	0.04	0.25	0.11	0.24	T1D
2p21	*THADA*	ILMN_1811624	847	0.08	0.1	0.77	0	0.09	0.2	0.16	0.06	0.98	0.22	CRO
2p16.1	*AHSA2*	ILMN_1798308	970	1	0	1	0	0.32	0.02	0.11	0.05	0.08	0.06	MS
			899	1	0	1	0	0.29	0.03	0.11	0.11	0.08	0.08	PS

Continued on next page

Supplementary Table 1 – continued from previous page

Region	Gene	Probe	SNPs	B cells		Monocytes		IFN24		LPS2		LPS24		Disease
				Assoc	Coloc	Assoc	Coloc	Assoc	Coloc	Assoc	Coloc	Assoc	Coloc	
			796	1	0	1	0	0.27	0.04	0.09	0.07	0.07	0.07	CEL
			984	1	0	1	0	0.33	0.03	0.11	0.06	0.08	0.06	UC
			980	1	0	1	0	0.33	0.03	0.11	0.07	0.08	0.06	CRO
	LOC339804	ILMN_1754501	970	1	0	1	0	1	0	1	0	1	0	MS
			899	1	0	1	0	1	0	1	0	1	0	PS
			796	1	0	1	0	1	0	1	0	1	0	CEL
			984	1	0	1	0	1	0	1	0	1	0	UC
			980	1	0	1	0	1	0	1	0	1	0	CRO
2p14	*SPRED2*	ILMN_1791232	403	0.09	0.48	0.92	0	0.92	0	0.92	0	0.92	0	CRO
	PLEK	ILMN_1795762	362	0.95	0.07	0.95	0	0.56	0.01	0.27	0.06	0.05	0.27	MS
	PPP3R1	ILMN_1796962	362	0.93	0	0.94	0	0.57	0	0.04	0.16	0.95	0	MS
2q12.1	*IL18R1*	ILMN_1781700	830	0.68	0.01	0.05	0.05	0.41	0.01	1	0	0.06	0.09	CEL
			978	0.78	0.3	0.05	0.09	0.42	0.02	1	0	0.07	0.08	CRO
	IL18RAP	ILMN_1721762	830	0.11	0.05	0.56	1.76	0.15	0.03	1	0	0.08	0.07	CEL
			978	0.13	0.06	0.39	0.05	0.18	0.05	1	0	0.13	0.07	CRO
2q31.3	*UBE2E3*	ILMN_2390338	746	0.99	0	1	0	0.87	0.03	1	0	1	0	CEL
2q37.1	*SP110*	ILMN_1813455	279	0.95	0	1	0	0.66	0	0.03	0.27	0.03	0.2	CRO
			293	0.91	0	1	0	0.35	0.01	0.03	0.27	0.04	0.19	MS
	SP140	ILMN_2246882	279	1	0	0.66	0.01	0.11	0.1	0.03	0.25	0.03	0.26	CRO
			293	1	0	0.63	0.05	0.11	0.05	0.03	0.24	0.03	0.26	MS
	ATG16L1	ILMN_1725707	520	1	0	0.15	0.04	0.99	0.03	0.15	0.16	1	0	CRO
		ILMN_2365881	520	1	0.01	1	0	1	0	0.87	0.17	1	0	CRO
2q37.3	*GPR35*	ILMN_1710221	186	0.03	0.71	1	0	1	0.15	0.06	0.65	0.03	0.59	UC
3p14.3	*ABHD6*	ILMN_1706344	704	0.33	0.02	0.9	0	0.91	0	0.91	0	0.91	0	RA
	DNASE1L3	ILMN_2049184	704	0.91	0	0.1	0.04	0.05	0.18	0.29	0.04	0.05	0.17	RA
	PDHB	ILMN_1739274	704	0.06	0.15	0.91	0	0.91	0	0.7	0	0.91	0	RA
	PXK	ILMN_1815063	704	0.36	0.02	0.91	0	0.91	0	0.09	0.08	0.16	0.04	RA
3q13.33	*KTELC1*	ILMN_1811104	373	0.55	0.01	1	0	1	0	0.59	0.02	1	0	MS
			309	0.51	0.01	1	0	1	0	0.55	0	1	0	CEL
			375	0.55	0.01	1	0	1	0	0.6	0.01	1	0	PBC
5p13.2	*IL7R*	ILMN_2342579	428	0.05	0.14	0.03	0.12	0.93	1.12	0.42	0.69	1	0	MS
			424	0.05	0.14	0.03	0.12	0.94	0.48	0.45	0.63	1	0	PBC
5p13.1	*PRKAA1*	ILMN_2357577	761	0.08	0.07	0.05	0.09	0.09	0.05	1	0	0.31	0.02	CRO
			692	0.07	0.09	0.04	0.09	0.09	0.05	1	0	0.09	0.12	MS
			699	0.06	0.13	0.04	0.11	0.09	0.16	0.92	0	0.08	0.12	UC
5q15	*CAST*	ILMN_1717234	950	1	0	1	0	1	0	0.98	0	1	0	PS
			862	0.34	0.07	0.88	0	0.88	0	0.14	0.19	0.88	0	CRO
		ILMN_1783627	950	0.13	0.04	1	0	1	0	0.95	0	1	0	PS
			862	0.11	0.1	0.88	0	0.88	0	0.81	0	0.86	0	CRO
	ERAP2	ILMN_1743145	950	1	0	1	0	1	0	1	0	1	0	PS
			862	0.9	0.19	0.9	0.23	0.9	0.23	0.9	0.23	0.9	0.23	CRO
	LNPEP	ILMN_1814737	950	0.59	0.03	1	0	0.99	0	0.5	0.01	1	0	PS

Continued on next page

Supplementary Table 1 – continued from previous page

Region	Gene	Probe	SNPs	B cells		Monocytes		IFN24		LPS2		LPS24		Disease
				Assoc	Coloc	Assoc	Coloc	Assoc	Coloc	Assoc	Coloc	Assoc	Coloc	
			862	0.63	0.7	0.91	0.35	0.89	0.25	0.5	0.24	0.91	0.33	CRO
5q21.1	*PAM*	ILMN_2313901	788	0.96	0	0.99	0	0.46	0.01	0.99	0	0.14	0.06	RA
5q31.1	*CSF2*	ILMN_1661861	743	0.08	0.12	0.07	0.26	0.98	0	0.09	0.14	0.08	0.19	UC
			822	0.08	0.12	0.07	0.2	0.98	0	0.09	0.1	0.08	0.11	PS
	P4HA2	ILMN_1795778	743	0.07	0.13	0.06	0.14	0.98	0	0.98	0	0.98	0.03	UC
			822	0.08	0.12	0.08	0.19	0.98	0	0.98	0	0.98	0	PS
		ILMN_2381697	743	0.09	0.12	0.06	0.19	0.98	0	0.98	0	0.98	0.02	UC
			822	0.1	0.11	0.06	0.15	0.98	0	0.98	0	0.98	0	PS
	SLC22A4	ILMN_1685057	743	0.1	0.14	0.99	0.35	0.97	0.04	0.09	0.11	0.08	0.12	UC
			822	0.1	0.12	0.98	0	0.97	0	0.1	0.2	0.09	0.14	PS
	SLC22A5	ILMN_1699357	743	0.98	0.01	0.98	0	0.98	0	0.95	0.01	0.89	0	UC
			822	0.98	0	0.98	0	0.98	0	0.96	0	0.89	0	PS
5q35.2	*CPEB4*	ILMN_1722025	611	0.96	1.32	0.97	2.19	0.97	2.09	0.97	2.02	0.97	2.17	CRO
			614	0.96	1.25	0.97	2.18	0.97	2.06	0.97	2.06	0.97	2.43	CRO
6q21	*TRAF3IP2*	ILMN_1701514	638	1	0	1	0	0.07	0.09	0.08	0.1	0.07	0.13	PS
			695	0.98	0	0.98	0	0.08	0.1	0.09	0.1	0.08	0.14	UC
6q25.3	*RSPH3*	ILMN_1788223	441	0.11	0.07	1	0	0.99	0	0.97	0	1	0	CEL
			515	0.12	0.08	1	0	0.99	0	0.97	0	1	0	CRO
			513	0.12	0.04	1	0	0.99	0	0.98	0	1	0	MS
6q27	*RNASET2*	ILMN_1671565	314	0.86	1.45	1	1.57	0.08	0.59	1	1.45	1	1.62	ATD
			487	0.69	0.01	1	0	0.09	0.28	1	0.01	1	0	CRO
	RPS6KA2	ILMN_1716218	314	0.06	0.09	1	0	0.96	0	0.99	0	1	0	ATD
			487	0.07	0.11	1	0	0.98	0	1	0	1	0	CRO
7p15.2	*SKAP2*	ILMN_1657129	720	0.95	0.75	0.05	0.13	0.09	0.06	0.05	0.1	0.08	0.12	CRO
			701	0.89	0.72	0.05	0.12	0.08	0.06	0.05	0.1	0.08	0.13	T1D
			734	1	0	0.06	0.1	0.17	1.3	0.07	0.27	0.09	0.09	MS
7p12.2	*FIGNL1*	ILMN_1778152	666	1	0.46	1	0.2	0.06	0.19	0.12	0.54	0.1	0.21	T1D
		ILMN_2389114	666	1	0.28	1	0.68	0.07	0.35	0.06	0.15	0.1	0.3	T1D
7q32.1	*IRF5*	ILMN_1670576	314	0.89	0.02	0.89	0.02	0.89	0.02	0.89	0.02	0.89	0.02	RA
			327	0.99	0.04	0.99	0.05	0.99	0.05	0.99	0.05	0.99	0.12	UC
			328	1	0	1	0	1	0	1	0	1	0	PBC
			315	0.89	0.02	0.89	0.02	0.89	0.02	0.89	0.02	0.89	0.02	RA
	TNPO3	ILMN_1683811	314	0.88	0	0.89	0	0.89	0	0.89	0	0.89	0	RA
			327	0.98	0.19	0.99	0.26	0.99	0.29	0.99	0.23	0.99	0.23	UC
			328	0.99	0	1	0	1	0	1	0	1	0	PBC
			315	0.88	0.01	0.89	0	0.89	0	0.89	0	0.89	0	RA
8p23.1	*BLK*	ILMN_1668277	349	**0.91**	**5.18**	0.02	0.36	0.03	0.21	0.1	2.75	0.03	0.63	RA
	C8orf13	ILMN_1687213	349	0.83	1.95	0.63	0	0.19	3.76	0.77	2.41	0.16	0.03	RA
8q21.12	FAM164A	ILMN_1789558	892	**1**	**3.52**	**1**	**3.12**	**1**	**3.61**	1	2.89	**1**	**3.17**	MS
		ILMN_2057981	892	**1**	**3.35**	**1**	**3.31**	**1**	**3.2**	0.9	2.34	1	2.98	MS
9p13.3	*GALT*	ILMN_1657475	393	0.24	0.04	0.99	0	0.81	0	0.12	0.09	0.09	0.14	RA
	IL11RA	ILMN_1664912	393	0.12	0.04	0.99	0	0.34	0.01	0.05	0.14	0.06	0.38	RA

Continued on next page

Supplementary Table 1 – continued from previous page

Region	Gene	Probe	SNPs	B cells Assoc	B cells Coloc	Monocytes Assoc	Monocytes Coloc	IFN24 Assoc	IFN24 Coloc	LPS2 Assoc	LPS2 Coloc	LPS24 Assoc	LPS24 Coloc	Disease
	SIGMAR1	ILMN_2398489	393	0.81	0	0.99	0	0.3	0.01	0.04	0.14	0.16	0.2	RA
9q34.3	*CARD9*	ILMN_1712532	507	0.06	0.12	1	1.89	1	1.37	**1**	**6.25**	**1**	**3.83**	CRO
			505	0.07	0.13	1	1.98	1	1.6	**1**	**11.96**	**1**	**6.52**	UC
10p11.23	*MAP3K8*	ILMN_1741159	124	0.95	0	0.95	0	0.82	0.66	0.82	0.93	0.02	0.43	CRO
10q21.1	*CISD1*	ILMN_2122952	538	1	0	1	0	1	0	1	0	1	0	CRO
		ILMN_2122953	538	0.74	0.01	1	0	1	0	1	0	1	0	CRO
10q21.2	*ADO*	ILMN_1690352	417	0.97	1.66	0.99	0.01	**0.96**	**4.88**	1	0	0.39	0.07	CRO
			402	0.93	0	0.98	0	0.81	0.01	1	0	0.36	0.04	UC
11q23.3	*C11orf60*	ILMN_1677080	338	0.03	0.23	0.13	0.09	0.85	0	0.19	0.03	1	0	CEL
			426	0.04	0.19	0.17	0.13	0.87	0	0.23	0.02	1	0	MS
	TMEM25	ILMN_1651745	338	1	0	0.03	0.2	0.05	0.13	0.05	0.15	0.04	0.27	CEL
			426	1	0	0.04	0.21	0.05	0.11	0.07	0.13	0.04	0.25	MS
	TRAPPC4	ILMN_1814650	435	0.43	0.01	1	0	1	0	0.2	0.05	0.98	0	MS
12p13.31	*LTBR*	ILMN_1667476	49	0.01	1.46	1	0	1	0	1	0	1	0	MS
			50	0.01	1.43	**1**	**8.52**	**1**	**3.65**	**1**	**4.43**	**1**	**4.65**	PBC
	CLEC2D	ILMN_1693781	580	1	1.84	0.05	0.22	0.05	0.11	0.08	0.12	0.05	0.16	MS
			552	0.91	0.04	0.04	0.11	0.04	0.11	0.08	0.32	0.04	0.15	T1D
	CLECL1	ILMN_1782729	580	1	0.06	1	0.18	1	0.09	1	0.22	1	0.22	MS
			552	0.92	0.17	0.94	0.63	0.94	0.63	0.94	0.58	0.94	0.57	T1D
	LOC728715	ILMN_1749875	580	0.07	0.09	0.1	0.25	0.05	0.12	1	0	0.06	0.19	MS
			552	0.07	0.16	0.07	0.1	0.04	0.12	0.9	0	0.05	0.11	T1D
12q13.2	*RPS26*	ILMN_2209027	258	1	0.65	1	0.65	1	0.65	1	0.66	1	0.65	T1D
12q14.1	*FAM119B*	ILMN_1723846	609	**1**	**3.57**	**1**	**4.05**	**1**	**3.72**	**1**	**3.33**	1	2.31	MS
	METTL1	ILMN_1815190	609	0.07	0.23	0.05	0.08	1	2.85	0.79	2.34	1	2.86	MS
	XRCC6BP1	ILMN_1767481	609	1	0	1	0	1	0	1	0	1	0	MS
12q24.31	*LOC728069*	ILMN_1668743	415	0.93	0	0.93	0	0.15	0.03	0.26	0.08	0.93	0	MS
13q14.11	*AKAP11*	ILMN_1693220	456	0.05	0.13	0.08	1.14	1	0	0.08	0.7	0.79	0	CRO
14q13.2	*FAM177A1*	ILMN_2287276	700	0.05	0.13	0.91	0	0.91	0	0.91	0	0.91	0	PS
14q13.2	*KIAA0391*	ILMN_1701512	700	0.52	0.01	0.91	0	0.07	0.22	0.07	0.27	0.91	0	PS
15q25.1	*CTSH*	ILMN_2390853	279	1	0	**1**	**99**	**1**	**96.12**	**1**	**95.19**	**1**	**99**	NAR
			325	1	0	**1**	**32.01**	**1**	**141.45**	**1**	**303.04**	**1**	**32.33**	T1D
16p13.13	*C16orf75*	ILMN_1790537	871	1	0	0.15	0.03	1	0	1	0	1	0	PBC
			884	1	0	0.16	0.03	1	0	1	0	1	0	MS
			866	1	0	0.16	0.02	1	0	1	0	1	0	T1D
			805	1	0	0.18	0.06	1	0	1	0	1	0	PS
			390	1	0	0.15	0.04	1	0	1	0	1	0	CRO
	DEXI	ILMN_1738866	871	0.41	0.02	1	0	1	0.02	1	0	1	0	PBC
			884	0.4	0.04	1	0	1	0.15	1	0	1	0	MS
			866	0.4	0.04	1	0	1	0.06	1	0	1	0	T1D
			805	0.26	0.03	1	0	1	0	1	0	1	0	PS
16p11.2	*ITGAX*	ILMN_2254635	486	0.07	0.1	0.98	0	0.07	0.25	0.05	0.13	0.05	0.11	PS
			344	0.07	0.15	0.98	0	0.06	0.21	0.04	0.34	0.04	0.25	ATD

Continued on next page

Supplementary Table 1 – continued from previous page

Region	Gene	Probe	SNPs	B cells		Monocytes		IFN24		LPS2		LPS24		Disease
				Assoc	Coloc	Assoc	Coloc	Assoc	Coloc	Assoc	Coloc	Assoc	Coloc	
	SEPHS2	ILMN_1687824	486	0.06	0.17	0.04	0.17	0.98	0	0.04	0.14	0.04	0.16	PS
16q12.1	*NOD2*	ILMN_1762594	181	0.02	0.44	1	0	0.28	0.02	0.96	0	0.13	0.04	CRO
	SNX20	ILMN_1798612	181	0.02	0.56	1	0	0.03	0.24	0.08	0.08	1	0	CRO
17q11.2	*KSR1*	ILMN_1754798	406	0.05	0.42	0.87	0	0.04	0.25	0.05	0.24	1	0	PS
	LGALS9	ILMN_1715760	406	0.14	0.08	1	0	1	0	1	0	0.1	0.1	PS
		ILMN_2412214	406	0.84	0	1	0	1	0	0.98	0	0.15	0.06	PS
17q12	*CRKRS*	ILMN_1707448	858	0.07	0.07	1	0	1	0	1	0	1	0	PBC
			868	0.07	0.07	1	0	1	0	1	0	1	0	CRO
			822	0.06	0.08	0.96	0	0.96	0	0.96	0	0.96	0	RA
			870	0.07	0.07	1	0	1	0	1	0	1	0	UC
	GSDMA	ILMN_1776115	858	0.07	0.09	0.06	0.08	1	0	1	0	1	0	PBC
			868	0.07	0.08	0.06	0.07	1	0	1	0	1	0	CRO
			822	0.06	0.08	0.06	0.08	0.96	0	0.96	0	0.96	0	RA
			870	0.07	0.08	0.06	0.07	1	0	1	0	1	0	UC
17q21.2	*CNTNAP1*	ILMN_1692398	392	1	0	0.05	0.33	0.03	0.2	0.03	0.2	0.05	0.13	MS
			397	1	0	0.05	0.19	0.03	0.25	0.03	0.2	0.05	0.12	CRO
			397	0.93	0	0.05	0.49	0.03	0.2	0.03	0.2	0.05	0.12	UC
19p13.3	*GPX4*	ILMN_1734353	179	0.95	0.15	1	0	0.03	0.27	0.05	0.14	0.04	0.19	CRO
		ILMN_2378952	179	0.63	0.23	1	0	0.04	0.18	0.03	0.25	0.04	0.2	CRO
19p13.2	*ICAM3*	ILMN_2212763	246	0.04	0.75	1	0	0.16	0.06	0.04	0.36	0.06	0.15	PBC
			258	0.04	0.53	1	0	0.16	0.05	0.05	0.34	0.06	0.14	CRO
			258	0.04	0.51	0.99	0	0.16	0.06	0.05	0.35	0.06	0.15	MS
			237	0.04	0.58	1	0	0.16	0.05	0.04	0.37	0.06	0.15	RA
			235	0.03	0.56	1	0	0.16	0.06	0.04	0.3	0.06	0.15	PS
			254	0.03	0.4	0.99	0	0.17	0.13	0.04	0.22	0.06	0.2	UC
			240	0.04	0.62	1	0	0.16	0.05	0.04	0.27	0.06	0.15	T1D
	ICAM4	ILMN_1681296	246	0.03	0.46	1	0	0.12	0.06	0.99	0	1	0	PBC
			258	0.03	0.39	1	0	0.1	0.08	0.99	0	1	0	CRO
			258	0.03	0.39	0.99	0	0.1	0.08	0.99	0	0.99	0	MS
			237	0.03	0.44	1	0	0.1	0.08	0.99	0	1	0	RA
			235	0.03	0.51	1	0	0.1	0.08	0.99	0	1	0	PS
			254	0.03	0.29	0.99	0	0.1	0.06	0.98	0	0.99	0	UC
			240	0.03	0.43	1	0	0.1	0.08	0.99	0	1	0	T1D
		ILMN_2393067	246	0.03	0.53	1	0	0.03	0.38	0.05	0.21	0.69	0	PBC
			258	0.04	0.52	1	0	0.03	0.34	0.05	0.21	0.71	0	CRO
			258	0.03	0.49	0.99	0	0.03	0.34	0.05	0.19	0.7	0	MS
			237	0.03	0.56	1	0	0.03	0.37	0.05	0.22	0.71	0	RA
			235	0.03	0.45	1	0	0.03	0.37	0.05	0.2	0.68	0	PS
			254	0.03	0.44	0.99	0	0.03	0.29	0.07	0.62	0.69	0	UC
			240	0.03	0.54	1	0	0.03	0.39	0.05	0.22	0.67	0	T1D
19p13.11	*IFI30*	ILMN_1807277	317	0.88	0.01	1	0	1	0	1	0	1	0	MS
	KIAA1683	ILMN_2158003	317	1	0	1	0	1	0	0.07	0.37	0.56	0.05	MS

Continued on next page

Supplementary Table 1 – continued from previous page

Region	Gene	Probe	SNPs	B cells		Monocytes		IFN24		LPS2		LPS24		Disease
				Assoc	Coloc	Assoc	Coloc	Assoc	Coloc	Assoc	Coloc	Assoc	Coloc	
20p13	*SIRPD*	ILMN_1769886	352	0.04	0.17	0.98	0	0.98	0	0.98	0	0.98	0	T1D
20q13.12	*PKIG*	ILMN_1702911	169	1	0	0.02	0.33	0.03	0.23	0.02	0.36	0.02	0.25	UC
		ILMN_1796627	169	1	0	0.03	0.19	0.02	0.32	0.04	1.41	0.02	0.42	UC
		ILMN_2406169	169	1	0	0.01	0.25	0.02	0.75	0.03	0.68	0.02	0.27	UC
	TTPAL	ILMN_2364131	169	0.97	0	1	0	0.04	0.15	0.02	0.29	0.94	0	UC
	CD40	ILMN_1779257	372	0.04	0.16	1	0	0.1	0.13	0.55	0.19	1	0	CRO
		ILMN_2367818	372	1	0	1	0	0.57	0.01	0.08	0.34	0.77	2.7	CRO
20q13.33	*TNFRSF6B*	ILMN_1661825	319	0.03	0.22	0.04	0.14	1	0	0.03	0.18	0.03	0.2	CRO
			319	0.03	0.38	0.03	0.21	0.87	0	0.03	0.39	0.03	0.28	UC
		ILMN_1794643	319	0.15	0.07	0.99	0.02	0.24	0.13	0.19	0.54	0.12	0.58	CRO
			319	0.12	0.05	0.87	0	0.2	0.03	0.12	0.06	0.08	0.15	UC
		ILMN_2183687	319	0.12	0.67	1	2.93	0.05	0.25	0.05	0.55	0.04	0.42	CRO
			319	0.08	0.21	0.89	0.17	0.04	0.17	0.04	0.28	0.03	0.23	UC
		ILMN_2331231	319	0.03	0.31	0.99	0	1	0	0.05	0.41	0.06	0.24	CRO
			319	0.03	0.27	0.88	0.05	0.87	0	0.04	0.32	0.06	0.3	UC
		ILMN_2331232	319	0.04	0.44	0.09	0.31	1	0	0.03	0.23	0.03	0.19	CRO
			319	0.03	0.26	0.08	0.22	0.87	0	0.02	0.27	0.03	0.34	UC
21q22.3	*C21orf33*	ILMN_1737588	173	0.12	0.11	0.92	0	0.03	0.21	0.03	0.44	0.03	0.19	UC
			191	0.13	0.12	1	0	0.03	0.19	0.03	0.4	0.03	0.17	CRO
22q12.2	*MTMR3*	ILMN_1803925	820	0.28	0.02	1	0	0.98	0	0.12	0.05	1	0	T1D
			850	0.3	0.05	1	0	0.98	0	0.13	0.13	1	0	UC
			780	0.3	0.04	1	0	0.98	0	0.12	0.11	1	0	CRO
	UCRC	ILMN_2366714	820	0.9	0.02	1	0	1	0	0.65	0.41	1	0	T1D
			850	0.91	0.01	1	0	1	0	0.59	0.01	1	0	UC
			780	0.9	0.01	1	0	1	0	0.58	0.01	1	0	CRO
22q13.1	*PDGFB*	ILMN_1737695	192	0.02	0.44	0.03	0.32	1	0.55	1	2.39	0.1	1.43	UC
			192	0.02	0.38	0.02	0.27	1	0	1	0	0.05	0.15	CRO
			191	0.03	0.66	0.03	0.4	1	0.73	1	0.46	0.1	1.28	PBC
		ILMN_1775822	192	0.02	0.46	0.01	0.46	1	0.18	1	0.17	1	0.19	UC
			192	0.02	0.44	0.01	0.43	1	0	1	0	1	0	CRO
			191	0.02	0.4	0.02	0.59	1	0.17	1	0.11	1	0.17	PBC
	SYNGR1	ILMN_1727805	192	1	0	1	0.13	0.02	0.39	0.02	0.45	0.07	0.56	UC
			192	1	0	1	0	0.02	0.4	0.02	0.43	0.06	0.17	CRO
			191	**1**	**13.93**	**1**	**11.96**	0.02	0.35	0.02	0.61	0.06	0.39	PBC
		ILMN_1810875	192	1	0	1	0	0.66	0	0.19	0.09	0.85	0	UC
			192	1	0	1	0	0.67	0	0.2	0.15	0.84	0	CRO
			191	**1**	**16.6**	1	0	0.68	0.01	0.19	0.1	0.83	0	PBC
